# Core-Shell Fe_2_O_3_@La_1−x_Sr_x_FeO_3−δ_ Material for Catalytic Oxidations: Coverage of Iron Oxide Core, Oxygen Storage Capacity and Reactivity of Surface Oxygens

**DOI:** 10.3390/ma14237355

**Published:** 2021-11-30

**Authors:** Hen Ohayon Dahan, Miron V. Landau, Roxana Vidruk Nehemya, Eran Edri, Moti Herskowitz, Chongyan Ruan, Fanxing Li

**Affiliations:** 1 Chemical Engineering Department, Blechner Center for Industrial Catalysis and Process Development, Ben-Gurion University of the Negev, Beer-Sheva 84105, Israel; henohay@post.bgu.ac.il (H.O.D.); roxana.vi@gmail.com (R.V.N.); edrier@bgu.ac.il (E.E.); herskow@bgu.ac.il (M.H.); 2 Department of Chemical and Biomolecular Engineering, North Carolina State University, 911 Partners Way, Raleigh, NC 27695-7905, USA; cruan3@ncsu.edu (C.R.); fli5@ncsu.edu (F.L.)

**Keywords:** core-shell morphology, iron oxide, lanthanum iron strontium perovskite, reactive oxygen species

## Abstract

A series of Fe_2_O_3_@LSF (La_0.8_Sr_0.2_FeO_3−δ_ perovskite) core-shell materials (CSM) was prepared by infiltration of LSF precursors gel containing various complexants and their mixtures to nanocrystalline aggregates of hematite followed by thermal treatment. The content of LSF phase and amount of carboxyl groups in complexant determine the percent coverage of iron oxide core with the LSF shell. The most conformal coating core-shell material was prepared with citric acid as the complexant, contained 60 wt% LSF with 98% core coverage. The morphology of the CSM was studied by HRTEM-EELS combined with SEM-FIB for particles cross-sections. The reactivity of surface oxygen species and their amounts were determined by H_2_-TPR, TGA-DTG, the oxidation state of surface oxygen ions by XPS. It was found that at complete core coverage with perovskite shell, the distribution of surface oxygen species according to redox reactivity in CSM resemble pure LSF, but its lattice oxygen storage capacity is 2–2.5 times higher. At partial coverage, the distribution of surface oxygen species according to redox reactivity resembles that in iron oxide.

## 1. Introduction

Core-shell nanocomposite materials in which nanoparticles of one solid (core) are covered with a shell of another mono-, polycrystalline or amorphous solid have been studied extensively over the last decade [1,2,3,4,5,6,7]. Application of the core-shell concept has increased the possibility to control surface chemical functionality by expanding the contact interface between solids with different composition and/or structure. Significant progress was reported for materials such as magnetized porous materials [1], materials for nanoelectronics [6], biocatalysts, bioadsorbents and biosensors [2,6] as well as heterogeneous catalysts in chemical reactions [3,4,5,7].

**Scheme 1:**
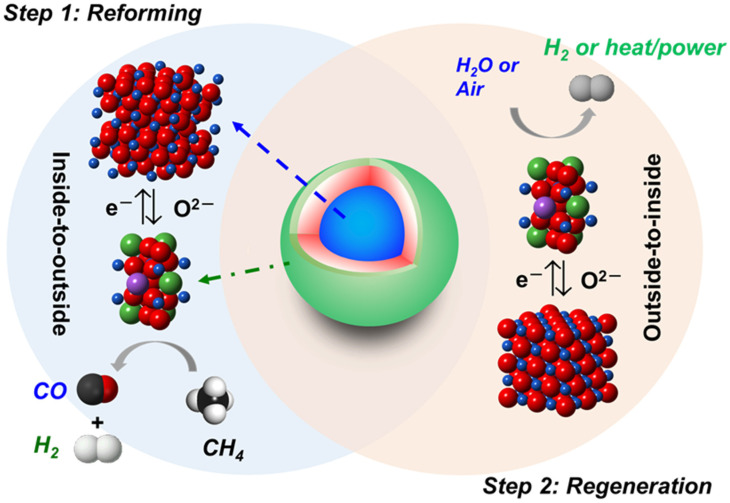
Schematic of core-shell redox catalyst functioning in a chemical looping mode partial methane oxidation reaction.

In heterogeneous oxidation catalysis, especially conducted in chemical looping mode using surface oxygens as active species for oxidative transformations of different substrates, application of core-shell materials may affect both the oxygen storage capacity and selectivity. For example, mixed oxides with perovskite structure are the most selective and highly regenerable catalysts for partial oxidation of methane to syngas [8] due to the proper states of oxygen ions on their surface [9,10]. However, they display relatively low oxygen storage capacity (OSC) of about 10 wt% compared with less active and less selective transition metal oxides such as Fe_2_O_3_ (30 wt%) [11,12]. Based on theoretical considerations of thermodynamics and diffusion of oxygen ions in mixed ionic-electronic conductive (MIEC) perovskites, Li and his group developed a model of perovskite@Fe_2_O_3_ core-shell redox catalyst predicting a positive synergistic effect of the formation of extended perovskite/iron oxide phase interface [13,14]. According to this model (Scheme 1) MIEC support facilitates countercurrent conduction of O^2−^ electrons, thereby allowing facile O^2−^ transport to and from the iron oxide irrespective of the porosity of the oxygen carrier particle. Interaction between the MIEC and iron oxide creates a solid-state O^2−^ and electron/hole pathway with complete accessibility of active oxygen in iron oxide. As a result, the iron oxide in the core-shell catalyst serves as a reservoir of such lattice oxygen at the perovskite shell. This is consistent with the high chemical potential of O^2−^ in iron oxides and facile transport of O^2−^ though the perovskite [12].

The oxygen ions delivered by the core reach the perovskite surface through the shell. The mechanism of bulk diffusion of ions in a proper solid assumes sequential occupation of vacancies in the atomic network by moving ions with the coordination corresponding to inherent structure of the parent solid [15,16,17,18]. Therefore, the state of oxygen ions that conducted through the perovskite shell is similar to that located at the pure perovskite surface. It creates a substantial increase of the OSC of core-shell material compared with pure perovskite along with the high activity-selectivity characteristics of perovskite shell.

Testing of core-shell materials prepared by insertion of iron oxide nanoparticles in colloidal precursors of La_0.8_Sr_0.2_FeO_3_ (LSF) perovskite demonstrated an increase of the catalytic activity of low-active Fe_2_O_3_ in oxidative methane conversion by nearly two orders of magnitude [13,19]. However, the products yield in partial oxidation of methane obtained with core-shell Fe_2_O_3_@LSF composite on weight basis was similar to pure active perovskite material [14]. Proper control of shell morphology and coverage of core nanoparticles (primary nanocrystals and their porous aggregates) is important for understanding the performance. Coverage (coating) of core particles with a continuous shell layer or decoration of core particles with separated nanoparticles of shell phase have been reported [20].

The information on coverage of different oxides with perovskites is limited. The Fe_2_O_3_ core surface covered with LSF perovskite by insertion of iron oxide nanoparticles to colloidal solution of LSF precursors was estimated to be 84%, based on surface fraction of Fe^x+^ ions measured by XPS [14]. Other characterization methods should confirm this value since the surface chemical composition measured by XPS differs significantly from the bulk composition of the material and may further change at the shell surface affecting the results. Sequential coating of Fe_2_O_3_ nanoparticles with Ti-hydroxide followed by its hydrothermal reaction with Pb-salt yielded a core-shell composite Fe_2_O_3_@PbTiO_3_ perovskite [21]. Based on TEM data, the core nanoparticles were covered with a continuous layer of Ti-hydroxide after the first step, while its further reactive crystallization to perovskite yielded segregation of the two phases, thus Fe_2_O_3_ was decorated with separate nanoparticles of perovskite with non-uniform and non-conformal coating.

Covering Sr_0.7_La_0.3_(Ti_1−x_Nb_x_)O_3_-perovskite with another perovskite La_x_Sr_y_Ba_z_Ce_ξ_O_δ_ shell was carried out by insertion of the former in colloidal solution of precursors of the latter, followed by high-temperature crystallization [22]. It also created segregation of the core and shell phases. According to FESEM-EDS analysis, the Ce- and Ti- mapping fields did not match the same areas as indicated by SEM micrographs. Preparation of dual perovskite CoFe_2_O_4_@BaTiO_2_ core-shell material by insertion of Co-ferrite nanoparticle to the citrate gel of Ba-Ti salts precursors with subsequent crystallization yielded a mixture of segregated nanoparticles of both perovskites [23]. This is clearly visible on TEM micrographs due to different contrast of the two phases. Solvothermal treatment of 9 nm CoFe_2_O_4_ perovskite nanoparticles in solution of MnC_l2_-Fe(acac)_3_ salts in oleic acid- oleyamine-trioctylamine complexants yielded 3 nm layer of MnFe_2_O_4_ ferrite [24]. Part of the CoFe_2_O_4_ surface was not covered, probably due to the relatively low content of MnFe_2_O_4_ in the composite.

A thick (400–600 nm) continuous layer of SrTiO_3_ perovskite was formed on Ti nanoparticles, prepared by hydrothermal treatment of the core powder in solution of Sr(OH)_2.8_H_2_O_25_ [25]. This reduces the efficiency of core-shell system in catalytic oxidations due to long path for diffusion of oxygen ions. In addition, a transition zone between core and shell of about 200 nm was found in this material, containing Ti, perovskite and unknown phases that may strongly affect the diffusion of ions in the shell. Contamination of the uniform perovskite shell with core components was observed also in Mg-Al hydrotalcite@LaNiO_3_ prepared by deep coating of hydrotalcite particles with aqueous solution of La- and Ni-salts followed by high-temperature calcination [26]. Formation of Fe@La_0.6_Sr_0.4_Fe_0.8_Al_0.2_O_3−δ_ perovskite core-shell material was reported by in situ encapsulation of metallic iron nanoparticles during thermochemical production of syngas [27]. This is a result of dynamic structural transformation between the parent perovskite phase and Fe@La_0.6_Sr_0.4_Fe_0.8_Al_0.2_O_3−δ_ core-shell composite during redox cycling in chemical looping mode. Fe nanoparticles were formed in the subsurface layer of perovskite crystals during the reduction. The complete coverage of core nanoparticles with a thin continuous perovskite layer confirmed by HRTEM explains the catalytic action of perovskite phase. However, this preparation strategy cannot be implemented for controlled synthesis of Fe_2_O_3_@perovskite core-shell materials.

Preparation of thin continuous perovskite layers on iron oxide particles with a proper control of core surface coverage is challenging. Many studies were carried out for identification of surface oxygen species in mixed oxides including perovskites of different compositions and their reactions with catalytic substrates [8,9,10,28,29,30]. The effects of surface coverage of oxygen carriers on the oxidation state-distribution-redox reactivity of oxygen atoms at the surface of perovskite shell were not addressed.

This study investigates Fe_2_O_3_@LSF core-shell material with different degrees of shell coverage and characterizes the amount and state of reactive oxygens compared with both core and shell pure components. Core-shell materials were prepared by infiltrating the perovskite precursors to the porous nanocrystalline aggregates of hematite with high pore volume and surface area. The level of the coverage depended on the type of complexants and the content of the shell as evident in the microscopy study. The oxidation states of surface oxygens were characterized by XPS and their concentrations and reactivity in redox transformations by TPR-TGA-DTG methods.

## 2. Materials and Methods

### 2.1. Preparation of Materials

#### 2.1.1. Preparation of Pure LSF Perovskite

La_0.8_Sr_0.2_FeO_3_ perovskite was synthesized by the sol-gel method [31]. The perovskite precursors were dissolved separately in aqueous solution (in molar ratio) and heated to 50 °C on a hot plate. 3.5 g iron nitrate nonahydrate (Fe(NO_3_)_3_·9H_2_O, Fluka, >98%) was dissolved in 6 mL DI water, 2.9 g lanthanum nitrate pentahydrate (La(NO_3_)_3_·5H_2_O, Sigma- Aldrich, 99.99%) was dissolved in 6 mL DI water, and 0.4 g strontium nitrate (Sr(NO_3_)_2_, Fluka, >99%) was dissolved in 2 mL DI water. Once fully dissolved, the perovskite precursors were mixed and the complexants: 8.3 g citric acid (Sigma-Aldrich) and 4.9 g glycine (Sigma-Aldrich) were added to the solution (the ratio of moles complexants to total moles of perovskite precursors was 6.3). Then the mixed solution was heated to 80 °C on a hot plate until the gel formed. The material was dried overnight at 110 °C (5 °C/min), calcined in air at 200 °C (10 °C/min) for 2 h and then at 600 °C (5 °C/min) for 2 h.

#### 2.1.2. Fe_2_O_3_ Hematite Source 

Hematite Fe_2_O_3_ used as core in preparation of biphasic materials was purchased from NANOCAT^®^Superfine iron oxide (SFIO), MACH I manufacturer (King of Prussia, Pennsylvania, USA). It consists of porous aggregates of 3–10 nm Fe_2_O_3_ nanocrystals with a surface area of 229 m^2^/g. It was used-as-purchased without any pretreatment.

#### 2.1.3. Preparation of Fe_2_O_3_@LSF Core-Shell Materials

**Scheme 2:**
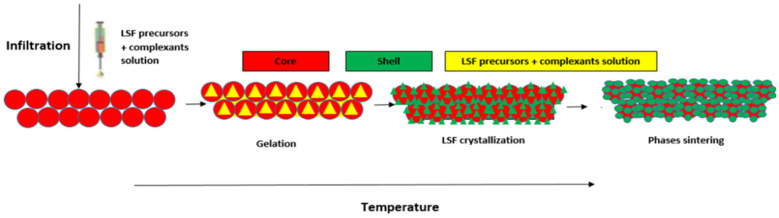
The infiltration strategy used for preparation of Fe_2_O_3_@LSF-perovskite core-shell materials.

The composite hematite/LSF materials were synthesized by the infiltration method [32,33,34]. This strategy (Scheme 2) involves insertion (infiltration) of aqueous solution containing perovskite precursors-nitrates of Fe, La and Sr at corresponding molar ratios and complexants (glycine, citric acid, ethylene glycol), to the pores of iron oxide core nanoparticles by incipient wetness impregnation followed by gelation inside the pores at elevated temperature.

After drying and calcination at 700 °C, the perovskite phase was formed and distributed at the outer surface of aggregates of hematite nanocrystals and in their pores. The infiltration-gelation was carried out at 60 °C while keeping the iron oxide powder on a hot plate. The ratio between the total moles of complexants (glycine, citric acid, and ethylene glycol) to the total moles of perovskite precursors (Fe, La, Sr) was 6.3. Materials CS-1 and CS-2 with 30 wt%. of perovskite phase and materials CS-3–CS-5 with 60 wt% of perovskite phase were prepared by controlling the weight ratio of total LSF in the added solution to the weight of iron oxide core.

CS-1 and CS-2 containing 30 wt% perovskite were prepared according to the following procedure. Aqueous solutions containing 14.4 mL DI water, perovskite precursors (0.2 g strontium nitrate, 1.8 g lanthanum nitrate pentahydrate and 2.2 g iron nitrate nonahydrate (Fe(NO_3_)_3_·9H_2_O, Fluka) and complexants (5.3 g citric acid with 3.0 g glycine in preparation of CS-1 catalyst; 5.3 g citric acid with 2.6 g ethylene glycol in preparation of CS-2 catalyst) were infiltrated to 3 g iron oxide by incipient wetness impregnation. Then the materials were dried overnight at 110 °C (5 °C/min). CS-1 material was calcined in air at 200 °C (10 °C/min) for 2 h and then at 600 °C (5 °C/min) for 2 h. Further, calcination was conducted consecutively at 700 °C for 3 h (5 °C/min). CS-2 material was calcined in air at 450 °C (5 °C/min) for 4 h and at 700 °C (5 °C/min) for 8 h.

The infiltration of metals-complexants solution in preparation of CS-3, CS-4, and CS-5 containing 60 wt% perovskite was carried out in two steps, due to the solubility limit of complexants. In the first step, aqueous solutions containing6.6 mL DI water, perovskite precursors (0.6 g strontium nitrate, 4.3 g lanthanum nitrate pentahydrate and 5.3 g iron nitrate nonahydrate) and complexants6.5 g citric acid with 3.8 g glycine in preparation of CS-3; 5.3 g glycine for CS-4 and 7.8 g citric acid for CS-5) were infiltrated into 2.0 g iron oxide. Then, the material was dried overnight at 110 °C (5 °C/min). In the second step, taking into account of the mass loss during drying, the materials obtained at the first step were infiltrated with aqueous solutions containing complexants (2.0 g citric acid with 1.2 g glycine dissolved in 13.9 mL DI water in preparation of CS-3; 3.0 g glycine dissolved within 14.0 mL DI water for CS-4 and 14.3 g citric acid dissolved in 24.6 mL DI water for CS-5). The materials were dried overnight at 110 °C (5 °C/min) and calcined at 200 °C (10 °C/min) for 2 h and then at 600 °C (5 °C/min) for 2 h. Further calcination was conducted at 700 °C (5 °C/min) for 2 h in case of CS-3 and CS-5 and for 10 h for CS-4 to complete crystallization of the perovskite phase. CS-6 was prepared as CS-3 inserting only Sr and La without iron precursor. In preparation of CS-7 all complexants were inserted at the first step and LSF precursors at the second step after drying. CS-8 was prepared as CS-3 inserting a 50 wt% excess of citric acid-glycine complexants. CS-9 was prepared as CS-5, the amounts of perovskite precursor and citric acid complexant were reduced to acquire 50 wt% loading of LSF-perovskite component.

### 2.2. Materials Characterization

The surface area, pore size, and pore volume of the catalysts were calculated from N_2_ adsorption-desorption isotherms using conventional BET (Brunauer–Emmett–Teller) and BJH (Barrett–Joyner–Halenda) methods, with NOVA 3200^e^ Quantachrome adsorption analyzer (Quantachrome, Anton Paar QuantaTec Inc., Boynton Beach, Florida, USA). Prior to analysis, the samples were outgassed under vacuum at 250 °C for 2 h. Conventional wide-angle XRD patterns were measured with a Panalytical Empyrean Powder Diffractometer (Cambridge, UK) equipped with a position-sensitive detector X’Celerator fitted with a graphite monochromator (Malvern Pananalytical Ltd, Malvern, UK), at 40 kV and 30 mA. The phases were analyzed with reference to the International Center for Diffraction Data (ICDD) database in HighScore software (Malvern Pananalytical Ltd, Malvern, UK). The phase content in catalytic materials was calculated using Rietveld refinement.

X-ray photoelectron spectroscopy (XPS) was conducted using X-ray photoelectron spectrometer ESCALAB 250 (Hamamatsu City, Japan) ultrahigh vacuum (1 × 10^−9^ bar) apparatus with an Al Kα X-ray source and a monochromator. Surface charge neutralization was performed using electron flood guns before the measurement. The charge neutralization was performed by calibration of the instrument with a carbon standard at a binding energy (BE) of 284.8 eV. The spectral components of O signals were found by fitting a sum of the single component lines to the experimental data by means of a nonlinear least-square curve fitting.

The Field Emission FE-SEM images were recorded on an SM-7400F (JEOL) instrument (JEOL USA Ink., Peabody, Massachusetts, USA). The morphology and in-depth analysis of individual nanoparticles were further investigated using the following field emission analytical transmission electron microscope. High resolution transmission electron microscopy (HRTEM) imaging was carried out using a JEOL JEM-2100F analytical TEM (JEOL USA Ink., Peabody, Massachusetts, USA) operated at 200 keV equipped with a GATAN 894 US1000 camera. Energy-filtered TEM (EFTEM) experiments were performed using a Gatan image filter (Gatan Inc. Pleasanton, California, USA). The lanthanum M-edge (832 eV) and iron L-edge (708 eV) were used for elemental mapping. Concentration profiles of the elemental distribution along nanoparticles were obtained using the line profile tool of Digital Micrograph software of Gatan Ink. (Pleasanton, California, USA) at characteristic energies of M- and L-edges of lanthanum and iron listed above. The samples for HRTEM were prepared by depositing a drop of ethanol suspension of the solid catalyst on a silica-coated grid. For imaging of particle’s cross-sections, slices with thickness of 30–50 nm were cut out from materials particles by the FIB (focused ion beam) technique using FEI Helios G4 UC instrument-Dual beam (SEM-FIB), (ThermoFischer Scientific, Waltham, Massachusetts, US). The slices were transferred to silicon TEM grid and characterized by EFTEM.

H_2_-TPR were recorded on Analyzed Autochem II 2920 (Micromeritics Co. Micromeritics Norcross, Georgia, USA). The detected TCD (thermal conductivity detector) signal intensity corresponding to H_2_ (the water was condensed by passing a cold trap before entering to detector) was calibrated with H_2_/Ar mixtures of different compositions. The samples (0.1 g) were heated under 8% H_2_/Ar (85 mL /min) flow with a heating rate of 10 °C/min from 70 to 870 °C and maintaining at the final temperature for 30 min. Non-isothermal TGA (Thermogravimetic analysis) was conducted under 4% H_2_/Ar flow of 80 mL/min at the temperature range from 250 to 870 °C (15 °C/min) followed by keeping at this temperature for 30 min. The measurements were performed using TGA Labsys EVO 1150 instrument.

## 3. Results and Discussion

### 3.1. Structure and Morphology of Synthesized Core-Shell Materials

Forming of a distinct crystal structure of multicomponent shell phase after infiltration of its precursors coating the core nanoparticles is a challenge. It requires a high-temperature crystallization step that generally leads to segregation of core and shell phases, preventing a uniform coating. In the present work, our synthetic strategy to overcome this challenge was twofold: i. use different complexing agents that strongly interact with the iron oxide surface; ii. confine the LSF precursors in the pores of the iron oxide in the first gelation step. These steps minimize phase segregation at the subsequent high temperature crystallization step. The pores of high surface area nanocrystalline aggregates of iron oxide with pore volume of ~1 cm^3^/g were filled with a solution of metal salts (La, Sr, Fe) and complexants followed by gelation at 60 °C. The gel was uniformly distributed along the core surface and in the pores due to co-complexation of dissolved metal ions with ions at the surface of iron oxide nanocrystals. Two processes were expected to proceed at the high-temperature LSF perovskite (LSF) crystallization step: sintering of iron oxide particles reducing the pore volume and growing of LSF shell crystallites. Confining LSF precursors inside iron oxide pores reduced the segregation of Fe_2_O_3_ and LSF phases preferring coating and formation of Fe_2_O_3_@LSF core-shell material with highly conformal coating.

**Figure 1:**
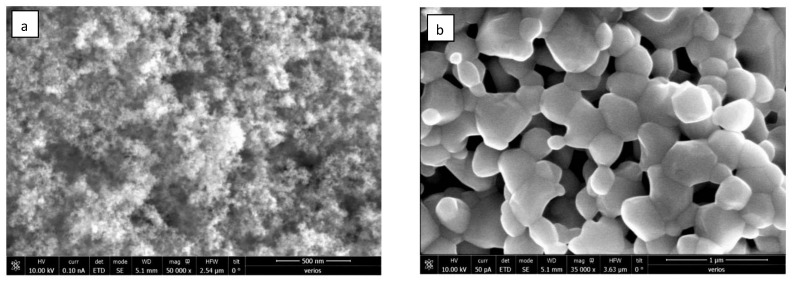
FE-SEM micrographs of parent Fe_2_O_3_ core material fresh (**a**) and after calcination at 700 °C (**b**).

The pure perovskite phase synthesized by co-gelation of La-Sr-Fe salts in aqueous solution in presence of citric acid (CA) and glycine (G) complexants was completely crystallized at 600 °C. Its XRD patterns correspond well to La_x_Sr_1−x_FeO_3_ (ICDD card #35-1480) with an average crystal size of 25 nm. The crystallization of LSF from the dissolved complex-metal ion precursors in iron oxide pores was studied separately from the behavior of core material. In addition, the iron oxide core particles stability was investigated in the 275–1000 °C range. The parent iron oxide material with XRD patterns shown in Figure S1, contained two types of hematite nanoparticles (ICCD card #33-664): well-crystallized (10 nm) and nanocrystals (<4 nm) characterized by wide reflections. The results listed in Table 1 show a gradual decrease of surface area and pore volume with increasing the temperature. The sintering of primary crystallites as shown in FE-SEM micrographs presented in Figure 1 agrees with the XRD data listed in Table 1 and Figure S1.

In nanocrystalline iron oxide, small crystals disappeared at temperatures > 300 °C. Primary crystals size increased from 50 nm at 700 °C to >>50 nm at 1000 °C. HRSEM micrographs shown in Figure 1 demonstrate a dramatic change of iron oxide texture at 700 °C compared with the parent material. The friable packed nanocrystals in parent material were converted to separated aggregates (pore volume decreased from 0.97 to 0.06 cm^3^/g) of 0.2–0.5 µm. Assuming a uniform coverage, the spherical core-shell model based on theoretical density of both phases gives complete covering of the Fe_2_O_3_ nanocrystal with 5–6 nm thick layer at 45–60 wt% LSF, as listed in Table 2. OSC calculated as OSC_CS_ = 0.1x + 0.3y, where x and y—weight fractions of LSF and Fe_2_O_3_ in the core-shell material and 0.1; 0.3—OSC_LSF_ and OSC_Fe_2___O_3__ (wt% O), respectively, decreases with the increasing LSF content.

**Figure 2:**
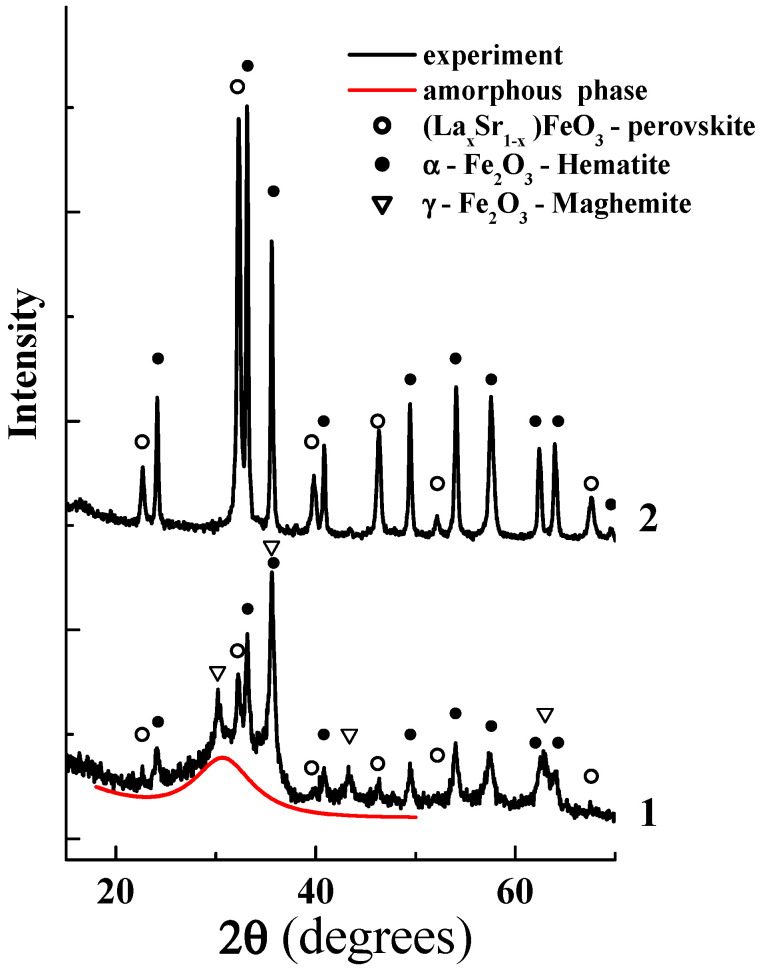
XRD patterns of 30 wt% La_0.8_Sr_0.2_FeO_3_/Fe_2_O_3_ after calcination for 3 h at 600 (**1**) 700 °C (**2**).

**Figure 3:**
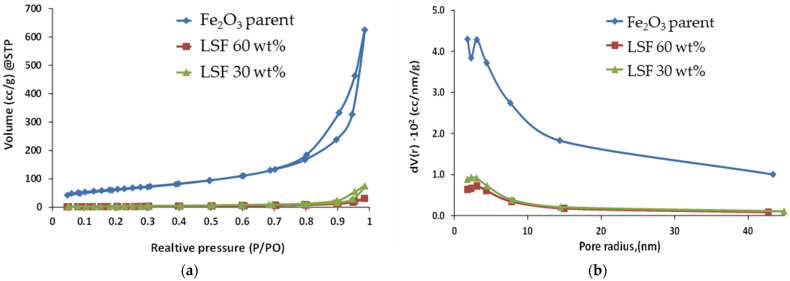
N_2_-adsorption-desorption isotherms (**a**) and pore size distributions (**b**) of fresh parent Fe_2_O_3_ and core-shell materials CS-1 and CS-3 containing 30 and 60 LSF-perovskite, respectively.

The preparation methods and properties of nine Fe_2_O_3_@LSF core-shell materials are listed in Table S1. The complete crystallization of LSF phase requires calcination temperature > 700 °C. This is indicated by the XRD patterns shown in Figure 2. The wide amorphous peak centered at 2θ ≈ 31°, representing disordered mixed La-Sr-Fe-oxide after calcination at 600 °C, completely disappears after raising the temperature to 700 °C. Interaction of parent core nanoparticles with LSF precursor induced partial recrystallization of hematite to γ-Fe_2_O_3_ maghemite phase (ICCD card #391346) (Table S1). As depicted in Figure 3, after insertion of 30–60 wt% LSF to the parent Fe_2_O_3_, a strong decrease of the pore volume was observed.

**Figure 4:**
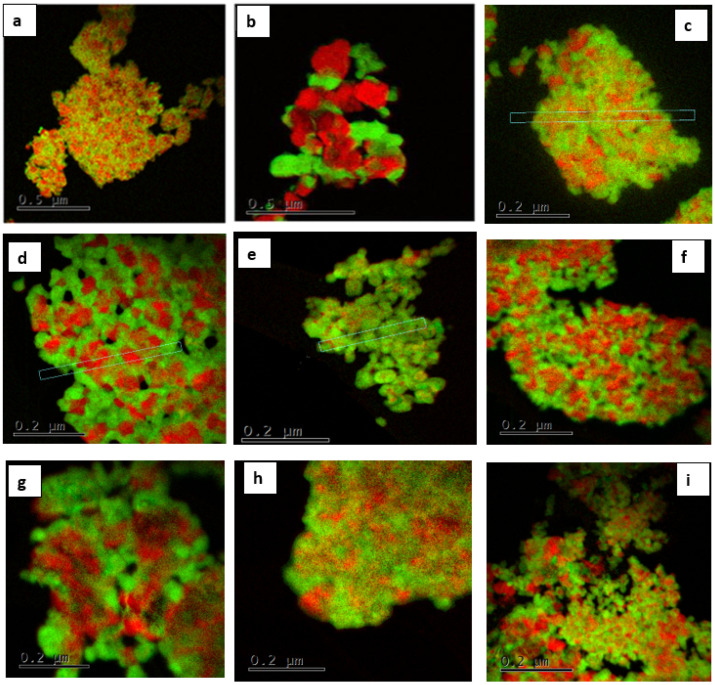
HRTEM-EELS images of Fe_2_O_3_@LSF core-shell materials: (**a**) CS-1; (**b**) CS-2; (**c**) CS-3; (**d**) CS-4; (**e**) CS-5; (**f**) CS-6; (**g**) CS-7; (**h**) CS-8; (**i**) CS-9. La-green; Fe-red.

The Fe and La elements in pure Fe_2_O_3_ and LSF materials (Fe-red, La-green) are different in HRTEM-EELS images (Figure S2). Therefore, the visual separation of core and shell phases in the particles is distinct, as shown in Figure 4. Application of glycine and citric acid as mixed complexant to prepare 30 wt% LSF core-shell particle CS-1 yielded a partial coverage of the core (Figure 4a). Using ethylene glycol in this mixed complexant scenario (CS-2) created a strong sintering and segregation of core and shell phases with a low contact interface between core and shell phases caused by poor core coverage (Figure 4b). The high-resolution TEM micrograph of CS-1 (Figure S3) shows that in contrast to CS-2, iron oxide nanocrystals are partially covered with LSF phase. Apparently, 30 wt% loading of LSF is not sufficient for complete coverage of core nanocrystals. Increasing the LSF loading to 60 wt% with the same mixed complexant (citric acid and glycine) significantly improved the conformality of the shell in CS-3 (Figure 4c).

60 wt% LSF materials using glycine (CS-4) and citric acid (CS-5) were prepared to further test the effect of the complexant on the core coverage. Glycine is more efficient than ethylene glycol combined with citric acid, as depicted in Figure 4b,d. LSF phase in CS-4 was uniformly distributed between Fe_2_O_3_ nanocrystals but the coverage was poor. Citric acid was the most efficient complexant, demonstrated in CS-5 (Figure 4e). The LSF phase was uniformly distributed along the particle, covering most of the iron oxide surface.

Amino acids such as glycine interacts with the surface of iron oxide, forming different binding states. It is adsorbed through carboxyl group (–COOH) in bridging coordination to iron ions, ionic coordination or by both amine and –COOH through surface hydroxyl groups of iron oxide [35]. The interaction of –COOH of glycine with the surface iron ions is significantly stronger than that of amino-group [36] and much stronger than H-bonding interaction with alcoholic hydroxyl of ethylene glycol. The interactions between Fe^2+^/Fe^3+^ ions and the O atoms of the carboxyl and carboxylate groups of the iron and glycine are dominated by electrostatic force [35]. Furthermore, the interactions between iron hydroxyls and glycine can occur with the amino group of glycine (H-bonding) or with the –COOH (ionic coordination) [35]. The ionic coordination interactions are stronger than the H-bonding. For both Fe^2+^ and Fe^3+^, their interactions with the –COOH of glycine are preferred. Glycine has one –COOH and citric acid has three.

CS-5 synthesized with citric acid (ratio of –COOH to perovskite metal precursors in solution was 18.8) yielded a better core coverage with LSF because of the stronger interaction between core surface and shell precursor than using citric acid combined with glycine (CS-3, the ratio of –COOH to perovskite metal precursors in solution was 8.4). Complete segregation occurred when using citric acid combined with ethylene glycol (CS-2) probably due to etherification of these two complexants. This strongly reduces the content of free –COOH in solution, leading to very weak interaction between iron oxide surface and LSF precursor.

Four other materials were synthesized and characterized to control the coverage of core nanoparticles. In all these materials the LSF phase was completely crystallized according to XRD analysis. Figure 4f indicates that this phase was located between nanocrystals of iron oxide rather than covering them. Using only complexants to facilitate their interaction with the core surface before inserting La-Sr-Fe-salts (CS-7) led to poorer coverage compared with insertion of complexants together with metal precursors and after them, as illustrated in Figure 4g,c. Increasing citric acid combined with glycine by 50% (CS-8) improved the coverage (Figure 4h,c) slightly. In this case the –COOH to perovskite metal precursors ratio was increased from 8.4 (in CS-3) to 12.6. CS-9 was prepared as CS-5 with 50 wt% LSF rather than 60 wt% LSF. Comparison of Figure 4i,e shows that decreasing of LSF content to <60 wt% reduced the extent of coverage.

**Scheme 3:**
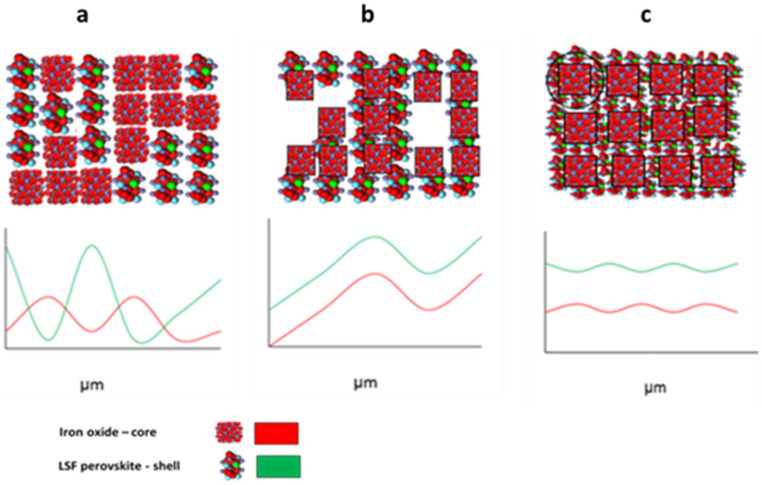
Effect of particles morphology on the Fe (red) and La (green) concentration profiles. (**a**) separate segregated two phases, (**b**) partially covered core particles containing wide pores, (**c**) completely covered core particles.

The morphology of core-shell particles the distribution of phases was further characterized using HRTEM-EELS imaging combined with FIB method. This in-depth analysis included La and Fe concentration profiles at selected directions. Signals intensities at concentration profiles reflect the local elements concentration in the bulk and the surface of core-shell particles. A model depicted in Scheme 3 accounts for three cases: (a) separate segregated two phases, (b) partially covered core particles containing wide pores, (c) completely covered core particles. In the first case, the minimum of Fe signal coincides with the maximum La signal and vice versa, reflecting the core or shell phases as a function of the distance from the particle edge. In the second, the minimum signals correspond to the vacant cages in the particle while in the third the signals move nearly in parallel, indicating that intracrystalline voids in the core are filled with the shell phase.

**Figure 5:**
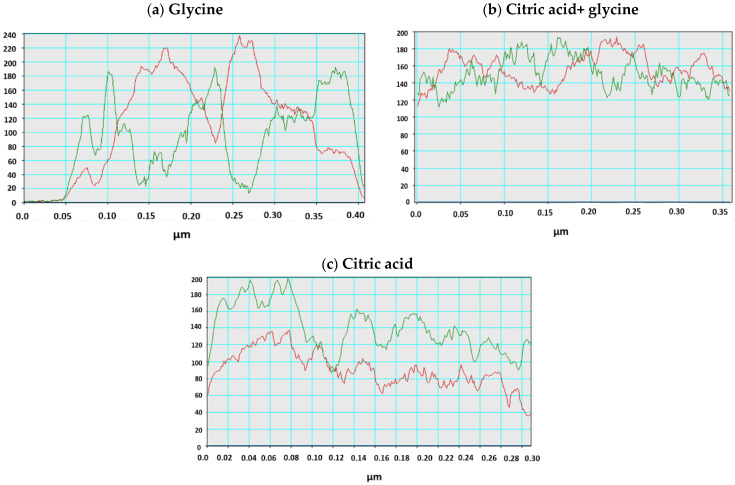
La- and Fe-concentration profiles of core-shell materials CS-4 (**a**); CS-3 (**b**) and CS-5 (**c**). La- green, Fe-red.

Figure 5 presents the concentration profiles of three materials CS-3, CS-4 and CS-5 prepared with different complexants. They were recorded in directions shown in Figure 4 by white lines. The La-Fe concentration profiles of CS-4 (Figure 5a) and CS-3 (Figure 5b) reflect a combination of high non-uniformity of LSF phase distribution with La- and Fe- rich regions, indicating a non-uniform distribution of shell phase. In CS-5, the La and Fe concentration profiles move in parallel (Figure 5c), suggesting a relatively uniform distribution. According to Scheme 3 the coverage of core iron oxide nanocrystals with LSF phase increases in the order of glycine (CS-4) < glycine combined with citric acid (CS-3) < citric acid (CS-5).

The percent coverage in CS-3–CS-5 materials were calculated based on the surface metals concentrations measured by XPS (Table 3). The details of calculation procedure are described in SM. Atomic concentrations of metal atoms only (La, Sr, Fe) in the surface layer obtained from XPS measurements were employed in the calculations, excluding concentrations of oxygen atoms. The sum of experimental atomic percentages of metal atoms was considered 100%, yielding the normalized concentrations values relative to this sum. Accordingly, Table 3 presents a comparison of the normalized bulk concentrations of the same metals measured by EDAX and normalized to 100% as described above. All three materials displayed similar chemical composition (72–75 wt% Fe) consistent with the content of LSF phase of about 60 wt%. However, according to XPS, the total iron concentration in the surface layer corresponding to the sum Fe_2_O_3_ core and LSF shell changes significantly from 61% in CS-4 to 46% in CS-5. It increases the surface coverage from 70% to 98%, in good agreement with estimations of relative core coverage according to HRTEM-EELS.

**Figure 6:**
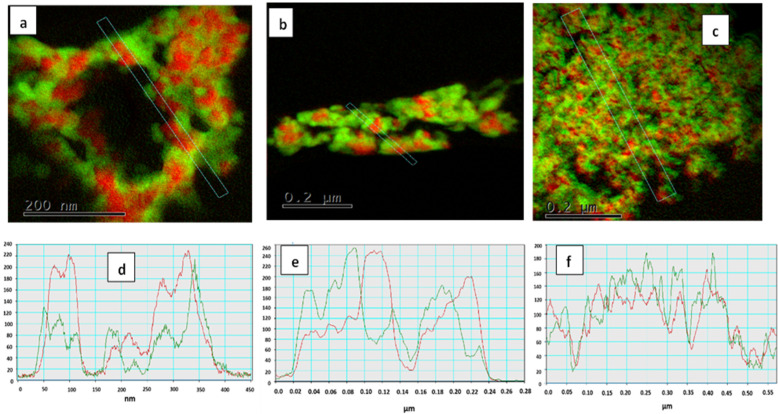
FIB-HRTEM-EELS images and La-, Fe-concentration profiles of slices from the particles of Fe_2_O_3_@LSF core-shell materials CS-4 (**a**,**d**), CS-3 (**b**,**e**) and CS-5 (**c**,**f**). La- green; Fe- red.

Thus, HRTEM-EELS images indicate an increase in the uniformity of the iron oxide core nanocrystals coverage with LSF shell (CS-4 < CS-3 < CS-5). The core-shell structures were further characterized by FIB-HRTEM-EELS for imaging of particle’s cross-sections. They are illustrated in Figure 6 together with the corresponding La-Fe concentration profiles. As shown in Figure 6a,d, CS-4 contains wide voids in bulk and is characterized by a high extent of phases segregation. The particles of LSF are located between nanocrystalline aggregates of iron oxide core (Figure 6a,d). The distribution of core-shell phases in CS-3 containing much less voids is more uniform (Figure 6b,e). Relatively high uniformity of core-shell phases distribution was observed in CS-5 particles (Figure 6c,f). Both iron oxide and LSF phases are uniformity distributed along the particle with the highest contact interface.

**Figure 7:**
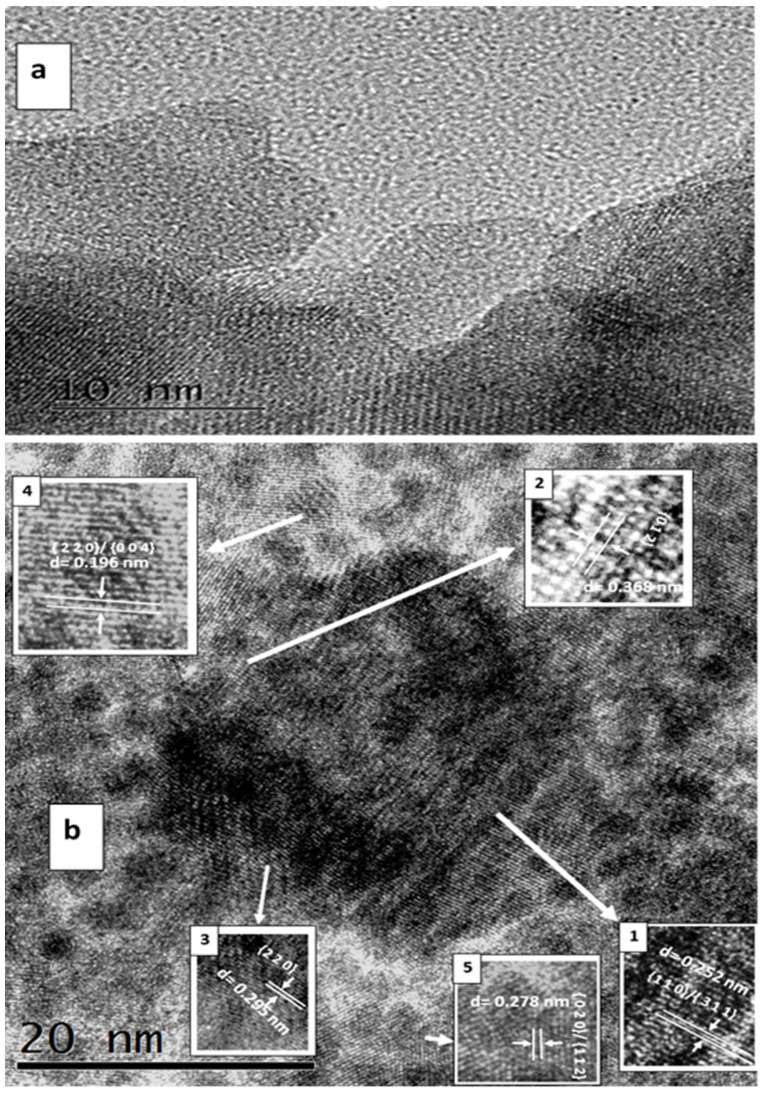
HRTEM images of LSF shell covering the of Fe_2_O_3_ nanocrystals in CS-4 (**a**) and CS-5 (**b**).

Additionally, the crystallinity of LSF shell was obtained from the high-resolution FIB-HRTEM images (Figure 7). Large patches of LSF phase in CS-4 are polycrystalline with disordered orientation (Figure 7a). The crystals are dense packed with dimensions larger than crystals domain size detected by XRD (30 nm). The LSF shell in CS-5 material consists of aggregates of 2–5 nm nanocrystals including the particles of iron oxide core (~50 nm). Parallel fringes (Figure 7b, inset 1) display a lattice spacing of 0.252 nm, which corresponds to (1 1 0) plane of rhombohedral phase hematite (ICDD Card No. 33-664) or to (3 1 1) plane of cubic phase maghemite (ICDD Card No. 39-1346). The lattice fringes observed in Figure 7b (inset 2) have a periodicity of 0.368 nm, which corresponds to (0 1 2) of rhombohedral phase hematite (ICDD Card No. 33-664). Figure 7b (inset 3) gives an interplanar distance periodicity of 0.295 nm which is associated with (2 2 0) plane of cubic phase maghemite (ICDD Card No. 39-1346). The above result indicates the core particles of CS-5 in core-shell materials consist of hematite (α-Fe_2_O_3_) and maghemite (γ-Fe_2_O_3_), in good agreement with XRD analysis (Table S1).

The crystallographic analysis of smaller nanocrystals with diverse orientation around the central core nanoparticle revealed that they have an orthorhombic structure of La_0.8_Sr_0.2_FeO_3_ corresponding to LSF shell. The fringes across images of these small nanoparticles have a periodicity of 0.196 nm (Figure 7b, inset 4) and 0.278 nm (Figure 7b, inset 5), which well match the d-spacing of (2 2 0) or (0 0 4) plane of La_0.8_Sr_0.2_FeO_3_ (ICDD Card No. 35-1480). As evident in Figure 7b, CS-5 presented a high contact interface between core and shell structure. The core and shell phases most probably intergrow along crystallographic plane of (1 1 0) for α-Fe_2_O_3_ and (1 1 0) for LSF, since for both crystals these planes are formed by rows of Fe atoms with low reticular density and suitable distances between M-O ions, as shown in Figure S5. CS-5 with near-complete coverage of LSF shell is expected to display at least twice higher OSC compared with pure LSF. However, the distribution of surface oxygen according to oxidation states and their reactivity at elevated temperatures would be close to that at the LSF surface. The surface oxygens of CS-4 with poorly covered core nanocrystals display the characteristic of iron oxide core, so OSC of this material is also expected to be high. To investigate the state-distribution of surface oxygens and their reactivity, XPS, H_2_-TPR and H_2_-TGA-DTG was employed.

### 3.2. Content-State-Distribution-Reactivity of Surface Oxygen Atoms in Hematite, LSF and Core-Shell Hematite@LSF

**Figure 8:**
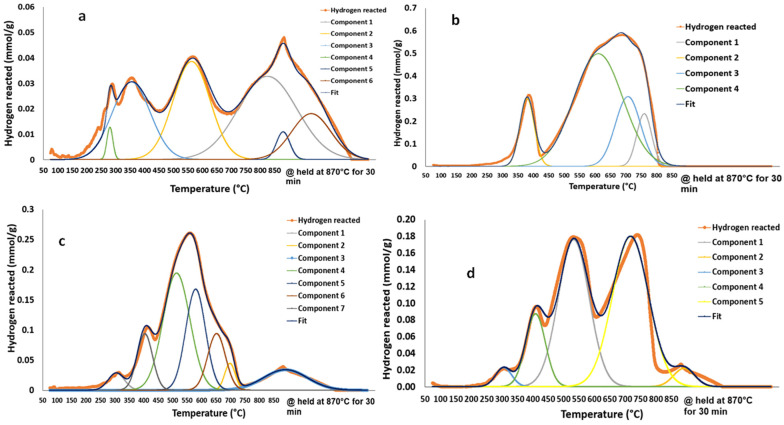
H_2_-TPR spectra recorded with LSF-perovskite (**a**), Fe_2_O_3_ (**b**), CS-4 (**c**), CS-5 (**d**).

**Figure 9:**
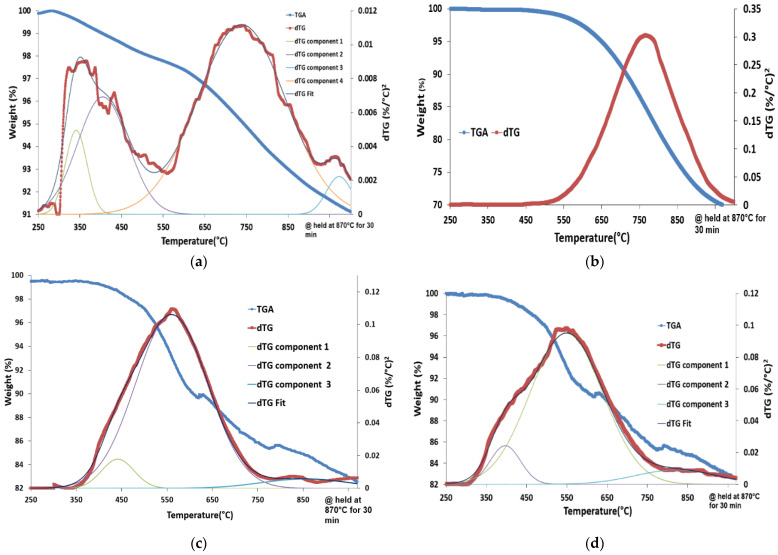
TGA-DTA thermograms for LSF (**a**), Fe_2_O_3_ (**b**) CS-4 (**c**), CS-5 (**d**).

The reactivity of oxygen in redox transformations was measured by H_2_-TPR. The corresponding spectra are presented in Figure 8 as envelops. The spectra were decomposed by deconvolution into components centered at temperatures given in Table 4. The number of components and their positions in corresponding spectra are determined by the type of material. Total amounts of reacted hydrogen and equivalent amounts of reacted oxygen removed as water (mmol/g material) representing the materials OSC are listed in Table 4. The amount of reacted oxygen in Fe_2_O_3_ was much higher than in LSF despite a smaller surface area. This is probably due to significant reduction to metallic state at 560–870 °C, while LSF remains mostly in a mixed oxide form (Table 5). The core-shell CS-4 and CS-5, as expected, displayed similar OSC, independent of the degree of core coverage. In both cases it was 2.5 times higher than LSF. The TGA-DTG results (Figure 9) are consistent with the H_2_-TPR measurements. The weight loss recorded with Fe_2_O_3_ and LSF materials representing their OSC was 30 wt% and 8 wt%, respectively, in agreement with their OSC reported in literature [11,12]. The total weight loss (OSC) of both core-shell materials was 17 wt%, 2.1 times higher than for LSF which correlates well H_2_-TPR results (Table 4).

The weight loss as a function of temperature (Figure 9) was observed over the high temperature range for Fe_2_O_3_ (550–900 °C) and over the entire temperature range for LSF (250–900 °C). The deconvolution of thermograms recorded with CS-4 and CS-5 core-shell materials yielded two peaks, one minor at about 400 °C and the other major at about 600 °C. The weight loss of CS-4 and CS-5 resembled LSF, though the intensity of the low-temperature peak in case of LSF was significantly higher and the high-temperature peak was shifted from 600 to 750 °C corresponding to lower reactivity. The above results indicate higher redox reactivity of oxygen belonging to core-shell materials compared with iron oxide core. LSF displays two types of oxygen with high and low reactivity. At the thermograms recorded with all materials the significant weight loss was observed at temperatures above 400 °C (Figure 9), while the hydrogen consumption in H_2_-TPR experiments started at about 300 °C. The relatively low hydrogen consumption at temperatures 300–400 °C in absence of weight loss may be attributed to its adsorption without water evolution as a first step of metals reduction leading to evolution of water at higher temperatures.

**Figure 10:**
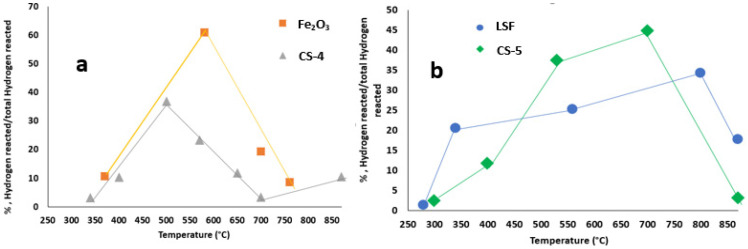
H_2_ consumption percentage as a function of temperature in H_2_-TPR: (**a**) - Fe_2_O_3_ and CS-4 materials; (**b**) LSF and CS-5 materials.

The amounts of reacted hydrogen as a function of temperature obtained by integration of intervals in Figure 8 are depicted in Figure 10. More than 60% of oxygen in Fe_2_O_3_ reacted at 580 °C. At lower and higher temperatures, the reacted oxygen is less than 20%, respectively. In LSF, it increased almost linearly between 300 and 750 °C. The distribution of oxygens according to their redox reactivity in core-shell materials was close to pure core for CS-4 with partial coverage of LSF (Figure 10a). On the other hand, the distribution of oxygen for CS-5 with high degree of core coverage resembled pure LSF (Figure 10b). So, the same total amount of reactive oxygen (OSC) in materials CS-4 band CS-5 is distributed in a significantly different ways among diverse oxygen states with different redox reactivity. The above results indicate that the complete coverage of core nanocrystals with perovskite shell is necessary to maintain the oxygen reactivity distribution while increasing the OSC compared with LSF.

**Figure 11:**
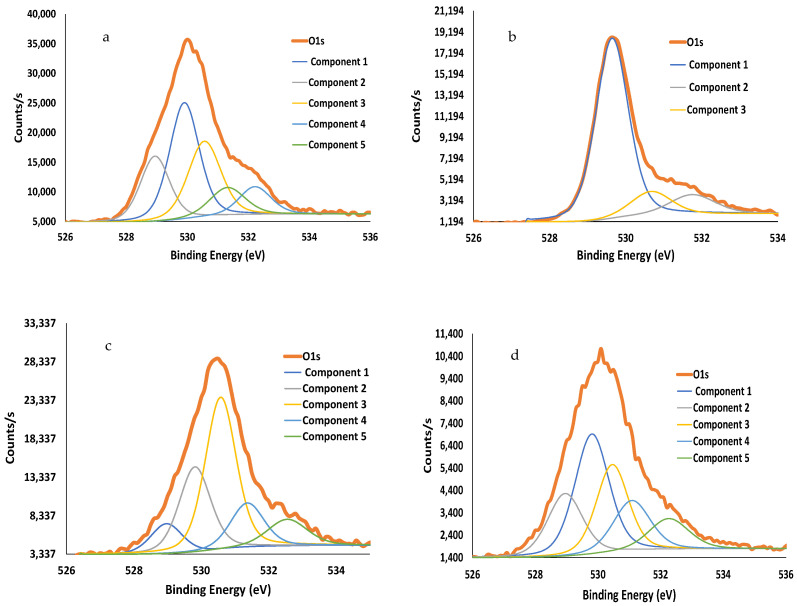
XPS spectra of O1s core: (**a**) LSF-perovskite; (**b**) Fe_2_O_3_ calcined at 700 °C and Fe_2_O_3_@LSF core-shell materials (**c**) CS-4 and (**d**) CS-5.

The XPS spectra of O1s core are depicted in Figure 11. The binding energies and their surface concentrations (at%) are shown in Table 6. The peak width (FWHM) of corresponding components obtained by fitting of XPS spectra are presented in Table S2. The XPS spectra of individual metal oxides [37] and mixed metal oxides such as perovskite (LSF) and perovskite-containing core-shell materials [28,38,39,40,41,42] include five groups of components that may be attributed to reactive surface oxygens (ROS). It comprises lattice oxygen O^2−^ (Lo), defect-affected O^2−^ V_M_, activated surface oxygen (Dao), activated surface oxygen resulting from oxygen adsorption: O_2(g_) → O^−^_2ads_ → O^2−^_2ads_ → O^−^_ads_ → O^2−^_surf_ (So), oxygen in carbonates and organic residual O=C (C_O_) and hydroxyl groups OH (OH_O_). At the surface of mixed oxides with perovskite structure are located lattice oxygens with BE < 530 eV related to reactivity of metal oxide and surface (adsorbed) oxygens including hydroxyl groups with high BE > 530 eV [28,41,42]. No organic and defect-affected oxygens were detected in Fe_2_O_3_. 10–15% C–O–C/O–C=O species in other materials may be a result organic complexants residual. The relative contributions of C–C/C–H and C–O–C/O–C=O components to the envelops of C1s spectra (Figure S6) shown in Table S3 correlate with the contributions of organic oxygen species in as-prepared materials (Table 6). This may be considered as confirmation for the ascription the O1s peak at BE 531.1–531.4 eV to organic oxygen species. All materials contain small amounts (9–11%) surface hydroxyls, which may be due to extensive surface dehydroxylation at 700 °C.

The distribution of oxygens in material CS-5 is very similar to LSF and significantly different from pure iron oxide. In LSF and CS-5, about 75–80% of oxygen is evenly distributed in the lattice, defect affected and surface states. Lattice oxygen is dominant in Fe_2_O_3_. This is consistent with H_2_-TPR and TGA-DTG results where oxygen with one specific reactivity was observed in Fe_2_O_3_ versus wide distribution of oxygen according to their redox reactivity for LSF and CS-5 (Figure 10). The partial coverage of iron oxide (CS-4) strongly reduces the contribution of lattice oxygen in the core, rendering surface oxygen a dominant state.

The XPS spectra of Fe2p core recorded with as-prepared materials are shown in Figure S7. The spectra of Fe_2_O_3_ displayed only one component with BE = 711.3 eV corresponding to Fe^3+^ ions in agreement with [43,44]. The Fe2p_3/2_ spectra of materials containing LSF perovskite phase include two components centered at 710.6–710.7 and 712.1–712.2 eV (Table S5). They correspond, respectively, to ions Fe^3+^ existing at the surface of iron oxide core and LSF perovskite phases and ions Fe^4+^ belonging to LSF [45,46,47]. In an ABO_3_ type perovskite such as LaFeO_3_, A-site substitution of the trivalent La^3+^ by the divalent Sr^2+^ causes oxidation of Fe^3+^ towards Fe^4+^. Increasing the contribution of higher energy component by passing from CS-4 to CS-5 reflects growing of core coverage with LSF in agreement with data of Figure 4 and Figure 6 and Table 3.

The O1s XPS spectra of spent materials from TPR experiments at 450, 650 °C and 870 °C are shown in Figure S5. The peak width (FWHM) of corresponding components obtained by fitting of XPS spectra are presented in Table S4. A comparison of different states contribution to the oxygen pool after removal of oxygen of high (450 °C), average (650 °C) and low (870 °C) redox reactivity is listed in Table 7. Increasing reduction temperature removes the less reactive oxygen and redistributes it. The phase composition of Fe_2_O_3_ core, pure LSF shell phase and two core-shell composite materials CS-4 and CS-5 is listed in Table 5.

The most reactive surface oxygen species in LSF (Lo), prone to redox transformations, were removed at 450 °C (Table 7). The remaining surface oxygen is mainly of DA_O_ type. Since the LSF phase does not decompose at 450–650 °C, this may be due to the formation of oxygen vacancies, which converts the Lo oxygen to DA_O_. A small amount (3–7 wt%) of metallic iron created at 450–650 °C does not contribute to the pool of oxygen species. Increasing the temperature to 870 °C leads to reductive decomposition, with only 15 wt% of oxygen-deficient LSF along with metallic iron, SrLaFeO_4_ (ICDD card # 29-1305) and La(OH)_3_ (ICDD card # 83-2034) phases (Table 5). LSF phase is converted from cubic perovskite to tetragonal SrLaFeO_4_ phase with the same content of all metal cations [48] that exhibits two peaks in O1s XPS spectra attributed to Lo and So types of oxygen [49]. Thus, Lo oxygen was identified in the O1s XPS spectra of LSF at 870 °C (Table 7) while So oxygen is not stable at this temperature. The high stability of Co oxygen in reductive condition increased their contribution to the surface oxygens pool due to removal of other oxygen types. OHo oxygen contribution decreased to 4% due to surface dehydroxylation then increased as La(OH)_3_ phase was formed at 870 °C. The La(OH)_3_ phase has relatively low thermal stability decomposing in inert atmosphere to La_2_O_3_ and water at 450 °C [50]. In the presence of water produced in H_2_-TPR, this phase is stable up to 900 °C [51,52].

The information about reactive oxygen species at the surface of iron oxides including Fe_2_O_3_ hematite (maghemite), Fe_3_O_4_ magnetite and FeO is limited [53,54,55,56,57]. The terminated stable surface planes of Fe_2_O_3_ contain L_O_ (Fe–O–Fe), ferryl Fe=O that can be considered as S_O_ and hydroxyl OH_O_ (Fe–OH, FeOOH) oxygen. This agrees with XPS data of iron oxide core presented in Table 6. The reduction of Fe_2_O_3_ in H_2_-TPR experiments proceeded in three steps with increasing temperature [58,59,60]: Fe_2_O_3_ → Fe_3_O_4_ → FeO → Fe^0^. The results depicted in Figure 8b are consistent with published phase transformations over temperature range. At 450 °C, the hematite phase completely disappeared forming magnetite (87 wt% Fe_3_O_4_, ICDD card # 19-629) which was partially converted to FeO (19 wt%, ICDD card # 6-615) and α-Fe^0^ (4 wt%, ICDD card # 6-696) (Table 5). It removed Lo, leaving So and OHo types of oxygen (Table 7), promoting the conversion of Fe_2_O_3_ to Fe_3_O_4_. Two types of oxygen were identified at the stable surface of Fe_3_O_4_, oxygen ions without subsurface tetrahedral iron ions and lattice oxygen species [53]. Their ratio is determined by the reduction level [56]. The O1s XPS spectra of Fe_3_O_4_ phase displayed two peaks centered at 530.3 and 532.1 eV, which are attributed to Lo (Fe–O–Fe) and OHo (Fe–O–H), respectively [61]. Reduction of hematite to magnetite shifted the BE of Lo type from 529.6 to 530.4 eV, reflecting a decrease of their redox reactivity (Table 6). While the OHo oxygens displayed a similar BE of 532.6 eV. After H_2_-TPR measurements at 650–870 °C, the Fe_2_O_3_ material was converted to metallic iron (Table 5). However, peaks were detected at 530.2–530.3 eV and 532.6–532.7 eV corresponding to So and OHo surface oxygen types (Table 6), which is probably due to adsorption of oxygen from air. Metallic iron adsorbs oxygen from atmospheric air that reconstructs the surface layer to a 2D FeO phase at high surface coverage [62,63]. The O1s XPS peak of FeO is located at 531.5 eV [64,65]. This indicates that the oxygen was adsorbed on crystals of Fe^0^ phase at low coverage being in the form of So and part of it in form of OHo.

The performance of LSF in core-shell CS-4 and CS-5 measured in H_2_-TPR was similar to that as pure phase (Table 7). It was stable at 450–650 °C and decomposed at 870 °C forming SrLaFeO_4_, La(OH)_3_ and Fe^0^ phases (Table 5). The difference was in the relative stability of Fe_2_O_3_ core which is more resistant to reductive transformations in CS-5. Higher content of LSF phase in CS-4 compared to CS-5 at 450–650 °C (Table 5) is induced by the deeper reduction of iron oxide core. The less protected iron oxide core in CS-4 lost more oxygen compared with CS-5. It is reflected by higher content of the intermediate (Fe_3_O_4_ and FeO) phases (8–32 wt% in CS-5 vs. 0–15 wt% in CS-4) which are not reduced to Fe^0^ at 450–650 °C.

The redox reactivity of surface oxygen in CS-5 resembled that of pure LSF with some differences attributed to its interaction with iron oxide. The main difference is the formation of DA_O_ species at 870 °C with no L_O_ and the removal and subsequent formation of OH_O_ species at 450–650 °C and 870 °C, respectively. The high oxygen mobility in SrLaFeO_4_ phase formed at 870 °C in presence of iron [66] may explain the existence of DA_O_ at 870 °C in contrast to LSF where this phase contributed to the surface oxygen pool with L_O_ oxygen. The second difference may be assigned to surface dihydroxylation of CS-5 at lower temperature and formation of La(OH)_3_ phase with OH_O_ oxygens only at 870 °C. The contribution of S_O_ and OH_O_ oxygen in CS-5 due to adsorption of atmospheric oxygen on metallic iron is negligible (Table 7).

For CS-4 with partial coverage of LSF, the redox reactivity of surface oxygens S_O_ > DA_O_ > L_O_ significantly differs from the fully covered CS-5 as well as pure reference core and shell materials. This may be due to the fact is that areas of core particles that are exposed at the external surface in CS-4 materials are covered with an atomic layer of La-Sr, changing the reactivity of surface oxygens.

For pure LSF and completely covered core-shell material (CS-5), comparison of reactive surface oxygen species removed at different temperatures in H_2_-TPR experiments showed similar nature and redox reactivity. Similar catalytic behavior of pure LSF and CS-5 is expected, with important differences. The oxygen storage capacity of CS-5 material is 2–2.5 times higher compared than pure LSF (Table 4, Figure 9), rendering the core-shell material potentially more active. In core-shell materials with partial coverage of LSF, the reactive oxygen species have different nature and reactivity compared with pure iron oxide.

## 4. Conclusions

Infiltration of LSF precursors with a range of complexants and their mixtures to nanocrystalline aggregates of hematite followed by thermal treatment was employed for preparation of core-shell materials. Optimization of various parameters was needed to crystallize the LSF shell with no phase segregation and achieve uniform core coverage. The content of LSF in composite material, temperature of thermal treatment and selection of complexant with high content of carboxyl groups were the important parameters. Near-complete coverage (98%) of the external surface of iron oxide core with LSF is achieved with 60 wt% LSF. At these conditions the shell phase is uniformly distributed also in the bulk of porous core-shell particles where relatively large 40–50 nm nanocrystals of iron oxide are surrounded by densely packed smaller LSF crystallites forming a polycrystalline shell. At a complete core coverage, the distribution of surface oxygens is similar to that in pure LSF, but the capacity of reactive oxygen was 2–2.5 times higher compared with LSF. At partial coverage, the distribution of surface oxygen species resembles that in iron oxide. However, the nature of reactive oxygen species is different.

## Figures and Tables

**Table 1:** Effect of calcination temperature on the texture parameters of pure hematite core.

Calcination Temperature, °C	25	275	300	350	600	700	800	1000
Content of hematite phases, wt%								
- well crystallized	74	81	97	100	100	100	100	100
- small nanocrystals < 4 nm	26	19	3	--	--	--	--	--
Crystal size of well crystallized hematite phase, nm	10	20	20	25	45	50	>50	>>50
Surface area, m^2^/g	229	127	99	50	45	8	2.7	1.0
Pore volume, cm^3^/g	0.97	0.64	0.42	0.23	0.1	0.06	0.01	0.004
Average pore diameter, nm	8.46	10.1	8.5	9.1	7.3	9.2	7.4	8.0

**Table 2:** Estimation of OSC and LSF-perovskite content in nanoparticle.

LSF Shell Thickness (nm)	LSF Content, wt%	Total OSC, wt% O
3	32	23.6
4	39	22.2
5	45	21.0
6	57	18.6

**Table 3:** Coverage of iron oxide core with LSF shell in synthesized core-shell materials according to XPS data.

Materials	Normalized Surface Concentrations of Metal Atoms, XPS, (at. %)			Normalized Bulk Concentrations of Metal Atoms (at. %)			Core Surface Coverage with LSF, %
	La	Sr	Fe	La	Sr	Fe	
Fe_2_O_3_	--	--	100	--	--	100	0
LSF	35	21	44	33	12	55	100
CS-4	20	19	61	22	6	72	70
CS-3	20	23	57	20	5	75	77
CS-5	40	15	46	21	5	74	98

**Table 4:** H_2_-TPR peaks positions, total amount of hydrogen and oxygen reacted.

Materials	Surface Area, m^2^/g	Total Amount of Reacted Hydrogen, (mmol/g)	Equivalent Amount of Removed Oxygen, (mmol/g)	TPR Peaks Positions, °C
LSF	17	2.4	1.2	280,340,560,800,870
Fe_2_O_3_	8	16.3	8.1	370,580,700,760
CS-4	11	6.1	3.0	340,400,500,570,650,700,870
CS-5	20	6.0	3.0	300,400,530,700,870

**Table 5:** Phase composition of Fe_2_O_3_, LSF, CS-4 and CS-5 after H_2_-TPR tests.

Material	Phase Composition: Phase/wt%.		
	After H_2_-TPR Up to 450 °C	After H_2_-TPR Up to 650 °C	After H_2_-TPR Up to 870 °C
Fe_2_O_3_	Fe3O4/87	Fe^0^/100	Fe^0^/100
	FeO/19		
	Fe0/4		
LSF		LSF/93 Fe^0^/7	LSF/15
	LSF/97		Fe^0^/25
	Fe0/3		La(OH)_3_/27
			SrLaFeO_4_/33
CS-4	LSF/72	LSF/70 Fe^0^/30	LSF/10
	Fe^0^/13		Fe^0^/46
	Fe_3_O_4_/8		La(OH)_3_/24
	FeO/7		SrLaFeO_4/_20
CS-5	LSF/63	LSF/66 Fe^0^/26 FeO/8	LSF/20
	FeO/19		Fe^0^/44
	Fe_3_O_4_/13		La(OH)_3_/18
	Fe^0^/5		SrLaFeO_4_/18

**Table 6:** Oxygen species detected by XPS in as-prepared materials.

Materials	Lattice Oxygen		Defect-Affected Oxygen		Surface Oxygen		Carbonate and Organics Oxygen		Oxygen in Hydroxyl Groups	
	B.E., eV	% at.	B.E., eV	% at.	B.E., eV	% at.	B.E., eV	% at.	B.E., eV	% at.
Fe_2_O_3_	529.6	75.7	-----	-----	530.7	12.5	----	-----	532.8	11.7
LSF	528.9	19.6	529.9	35.4	530.5	25.6	531.3	10.2	532.2	9.31
Core-shell CS-4	529.0	8.6	529.8	24.8	530.6	43.3	531.4	13.6	532.6	9.7
Core-shell CS-5	529.0	18.0	529.8	33.7	530.5	23.0	531.1	15.2	532.3	10.2

**Table 7:** Oxygen species detected by XPS after H_2_-TPR experiments.

Materials	Lattice Oxygen		Defect-Affected Oxygen		Surface Oxygen		Carbonate and Organics Oxygen		Oxygen in Hydroxyl Groups	
	B.E., eV	% at.	B.E., eV	% at.	B.E., eV	% at.	B.E., eV	% at.	B.E., eV	% at.
LSF fresh	528.9	19.6	529.9	35.4	530.5	25.6	531.3	10.2	532.2	9.31
H_2_-TPR 450 °C	-----	-----	530.2	35.4	-----	-----	531.9	51.8	533.7	12.8
H_2_-TPR 650 °C	-----	-----	529.5	70.5	-----	-----	531.7	25.6	532.7	3.93
H_2_-TPR 870 °C	528.7	29.2	-----	-----	-----	-----	531.1	57.3	533.4	12.8
CS-4 fresh	529.0	8.6	529.8	24.8	530.6	43.3	531.4	13.6	532.6	9.7
H_2_-TPR 450 °C	528.3	36.0	529.6	50.0	-----	------	531.0	14.0	-----	-----
H_2_-TPR 650 °C	528.9	36.5	530.1	43.2	-----	-----	531.6	20.3	-----	-----
H_2_-TPR 870 °C	529.2	35.5	----	----	----	-----	531.0	40.6	532.7	23.9
CS-5 fresh	529.0	18.0	529.8	33.7	530.5	23.0	531.1	15.2	532.3	10.2
H_2_-TPR 450 °C	-----	-----	529.4	48.9	530.3	38.3	531.8	12.7	-----	-----
H_2_-TPR 650 °C	-----	-----	529.4	87.7	-----	-----	531.5	12.3	-----	-----
H_2_-TPR 870 °C	-----	-----	529.6	45.2	-----	-----	531.4	40.1	533.0	14.6
Fe_2_O_3_ fresh	529.6	75.7	-----	-----	530.7	12.5	----	---	532.8	11.7
H_2_-TPR 450 °C	-----	-----	-----	-----	530.4	73.1	---	----	532.6	26.7
H_2_-TPR 650 °C	------	------	------	------	530.2	68.2	------	------	532.6	31.8
H_2_-TPR 870 °C	-----	-----	-----	-----	530.3	64.3	---	----	532.7	35.7

## Data Availability

The datasets supporting the conclusions of this article are included within the article.

## Supplementary Materials

The following are available online at https://www.mdpi.com/article/10.3390/ma14237355/s1, Calculation of % coverage of iron oxide core with LSF shell based of XPS measurements, Figure S1: XRD patterns of pure iron oxide core. Fresh: (1) hematite particles < 4 nm, well crystallized hematite (2); after calcination at 450 °C (3); after calcination at 700 °C (4), Figure S2: HRTEM-EELS images of pure Fe_2_O_3_ (a) and LSF (b) materials, Figure S3: HRTEM-EELS image of core-shell material CS-1 recorded at high magnification. La- green, Fe- red, Figure S4: XPS spectra of O1s core recorded with materials after H_2_-TPR-experiments at 450 °C, 650 °C and 870 °C: (a) LSF; (b) Fe_2_O_3_ (calcined at 700 °C); (c) CS-4; (d) CS-5, Figure S5: Atomic arrangements at the crystal planes (110) of α-Fe_2_O_3_ and LSF materials: (a) horizontal projection; (b) frontal projection, Table S1: Preparation methods and properties of materials after calcination in air at 700 °C. Figure S6: XPS spectra of C1s core: (a) LSF-perovskite; (b) Fe_2_O_3_ calcined at 700 °C and Fe_2_O_3_@LSF core-shell materials (c) CS-4 and (d) CS-5. Figure S7: XPS spectra of Fe2p_3/2_ core: (a) LSF-perovskite; (b) Fe_2_O_3_ calcined at 700 °C and Fe_2_O_3_@LSF core-shell materials—(c) CS-4 and (d) CS-5. Table S2: The peak width measured in deconvoluted XPS spectra of as-prepared materials presented in Figure 11. Table S3: The peak width measured in deconvoluted XPS spectra of materials recorded after H_2_-TPR testing and presented in Figure S4. Table S4: Carbon species detected by XPS in as-prepared materials. Table S5: Components of XPS spectra of Fe2p_3/2_ core recorded with as-prepared materials.
