# Supplementary material for: Core-Shell Fe2O3@La1−xSrxFeO3−δ Material for Catalytic Oxidations: Coverage of Iron Oxide Core, Oxygen Storage Capacity and Reactivity of Surface Oxygens
Source: Materials (Basel). 2021 Nov 30;14(23):7355. doi: 10.3390/ma14237355 (PMC8658574; doi:10.3390/ma14237355)
Supplement: Supplementary file 1 [file materials-14-07355-s001.zip › materials-1477198-supplementary.pdf]

materials

MDPI

Supplementary Materials:

Core-Shell Fe_2O_3@$La_{1-x}Sr_xFeO_{3-\delta}$ Material for Catalytic Oxidations: Coverage of Iron Oxide Core, Oxygen Storage Capacity and Reactivity of Surface Oxygens

Supplementary Materials

Calculation of percent coverage of iron oxide core with LSF shell based on metals surface concentrations measured by XPS .

The calculations of %coverage of iron oxide core surface with the LSF shell in core-shell materials was done based on XPS data about the atomic surface concentration of metals (La, Fe, Sr). It was assumed that at the surface of Fe_2O_3 core exist 100 FeO atoms (iron atoms belonging to the iron oxide surface) per 100 metal atoms. According to XPS data, at the LSF surface was detected 44 atoms Fe_P (iron atoms belonging to perovskite surface), at the surface of CS-3 material – 57 (Fe_O+Fe_P) iron atoms and in CS-4 material – 61 (Fe_O+Fe_P) atoms per 100 metal atoms and at the surface of CS-5 material – 46 (Fe_O+Fe_P) atoms per 100 metal atoms. Assuming that the surface of core-shell materials represents a sum of surfaces belonging to pure (not LSF-covered) iron oxide and pure LSF, the calculations were done looking for the balance of iron atoms. If the share of Fe atoms representing pure core (Fe_O) in core-shell material is x atoms per 100 atoms the amount of Fe_P atoms will be 0.44.(100-x) per 100 atoms. The atomic balance equation:

$$1 \times (Fe_O) + 0.44\ (100\text{-}x)\ (Fe_P) = Fe_{total} \quad (1)$$

where Fe_{total} – total amount of Fe atoms (Fe_O+Fe_P) per 100 metal atoms detected at materials surface by XPS, where Fe_{total} – total amount of Fe anoms (Fe_O+Fe_P) per 100 metal atoms detected at materials surface by XPS, thus %coverage = 100 – x.

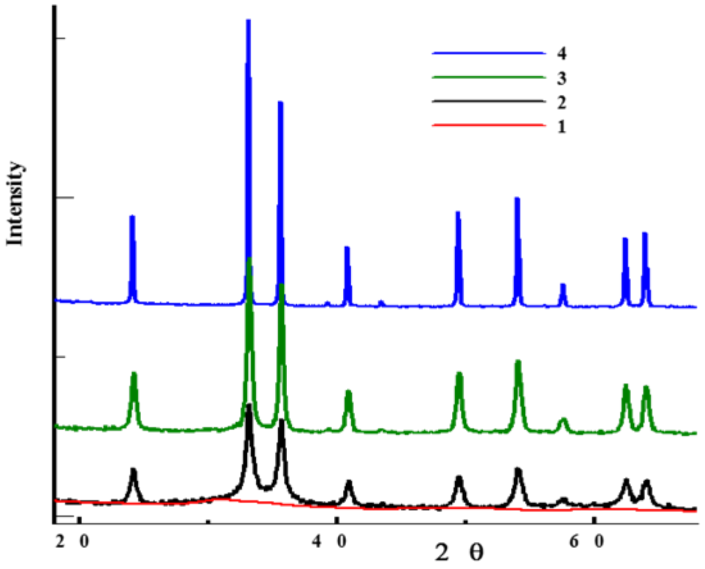

Figure S1. XRD patterns of pure iron oxide core. Fresh: **(1)** - hematite particles <4 nm, well crystallized hematite **(2)**; after calcination at 450°C **(3)**; after calcination at 700°C **(4)**.

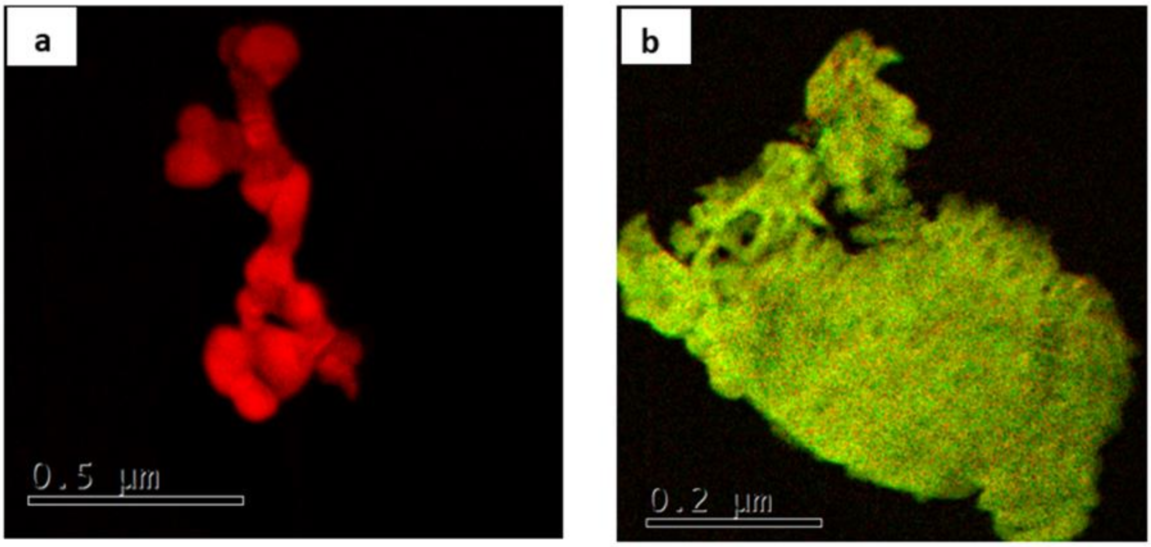

Figure S2. HRTEM-EELS images of pure Fe_2O_3 **(a)** and LSF **(b)** materials.

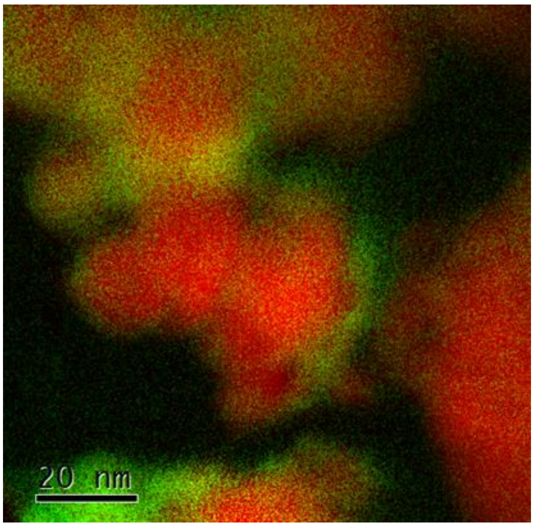

Figure S3. HRTEM-EELS image of core-shell material CS-1 recorded at high magnification. La - green, Fe – red.

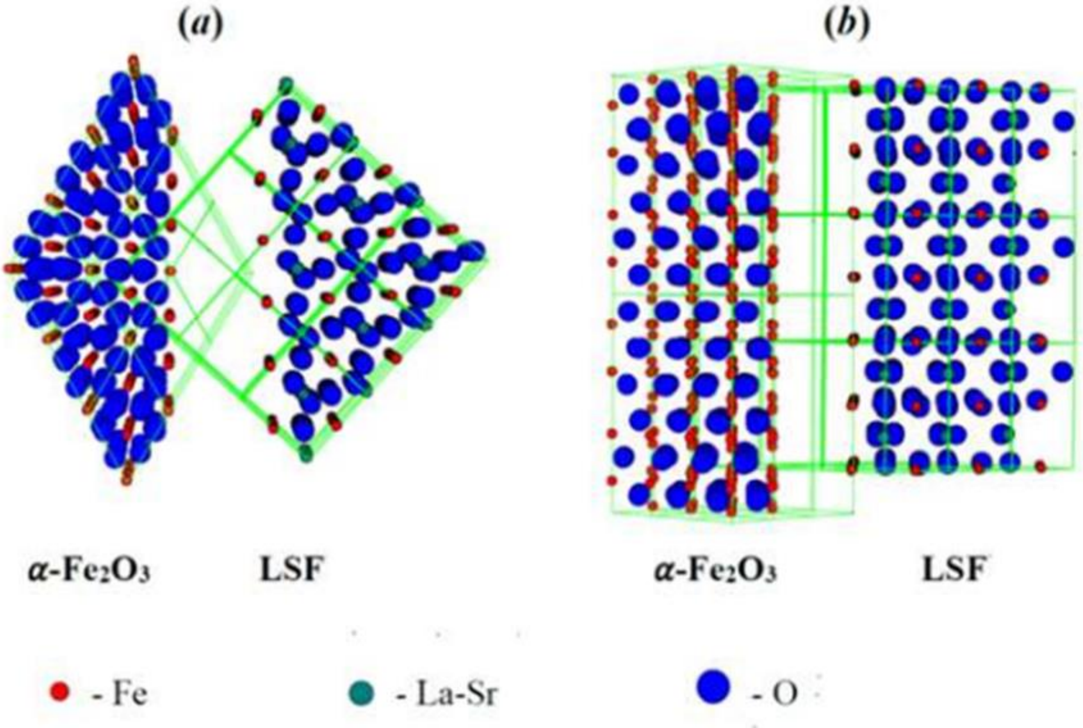

Figure S4. Atomic arrangements at the crystal planes (110) of α-Fe2O3 and LSF materials: (a) horizontal projection; (b) frontal projection.

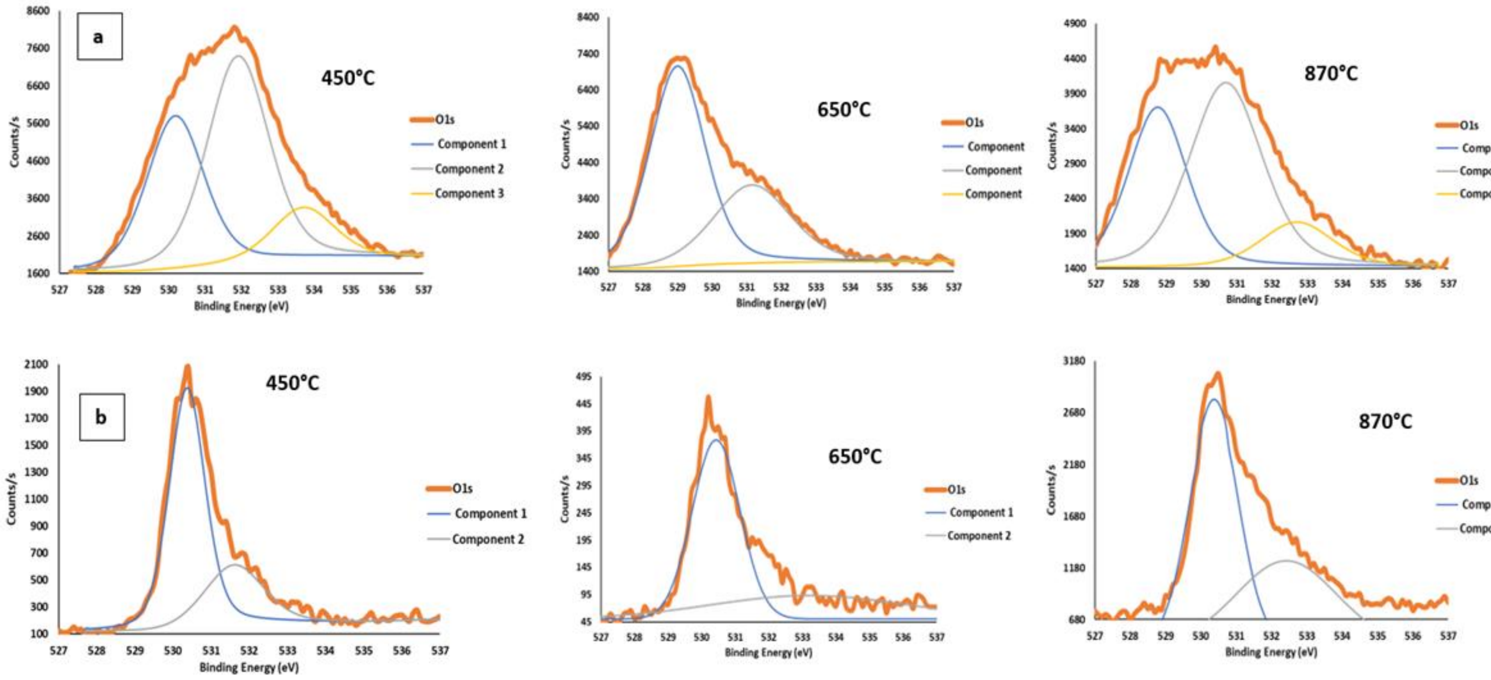

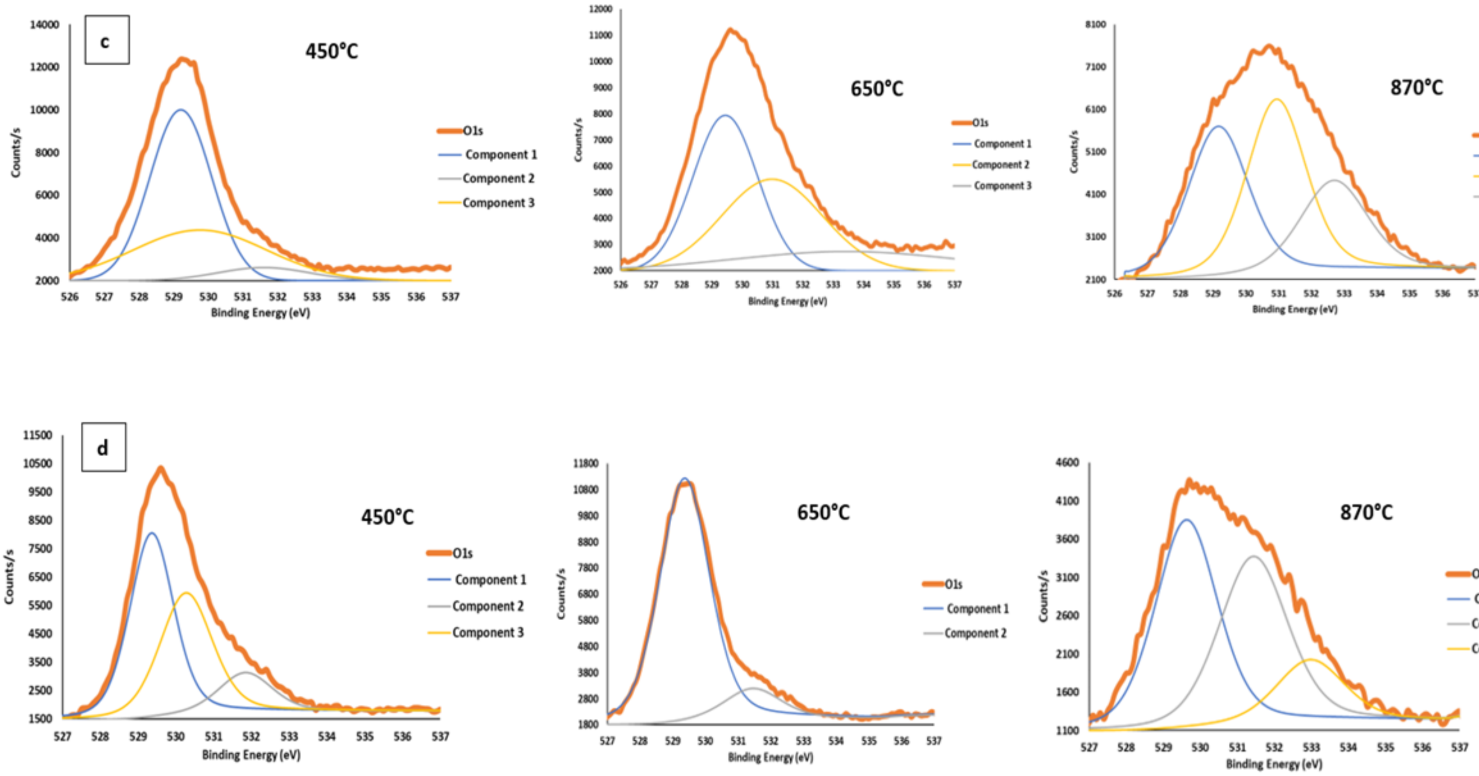

Figure S5. XPS spectra of O1s core recorded with materials after H_2-TPR-experiments at 450 °C, 650 °C and 870 °C: **(a)** LSF; **(b)** Fe_2O_3 (calcined at 700˚C); **(c)** CS-4; **(d)** CS-5.

Table S1. Preparation methods and properties of materials after calcination in air at 700 °C.

**Materials**	Preparation method
**Fe_2O_3**	Calcination at 700 ºC
$La_{0.8}Sr_{0.2}FeO_3$	Sol-gel synthesis, citric acid-glycine complexants
CS-1	Infiltration of aqueous solution of Sr-La-Fe salts and citric acid -glycine complexants to fresh not calcined Fe_2O_3 powder, gelation drying, calcination
CS-2	Infiltration of aqueous solution of Sr-La-Fe salts and citric acid -ethylene glycol complexants to fresh not calcined Fe_2O_3 powder, gelation drying, calcination
CS-3	Infiltration of aqueous solution of Sr-La-Fe salts and citric acid – glycine complexants to fresh not calcined Fe_2O_3 powder, gelation drying, calcination
CS-4	Infiltration of aqueous solution of Sr-La-Fe salts and glycine complexant to fresh not calcined Fe_2O_3 powder, gelation drying, calcination
CS-5	Infiltration of aqueous solution of Sr-La-Fe salts and citric acid complexant to fresh not calcined Fe_2O_3 powder, gelation drying, calcination
CS-6	Infiltration of aqueous solution of Sr-La salts and citric acid – glycine complexants to fresh not calcined Fe_2O_3 powder gelation drying, calcination
CS-7	Infiltration of citric acid – glycine complexants solution to fresh not calcined Fe_2O_3 powder, drying, infiltration of Sr-La-Fe salts solution, gelation drying, calcination
CS-8	Infiltration of aqueous solution of Sr-La-Fe salts 50% excess of citric acid – glycine complexants to fresh not calcined Fe_2O_3 powder, gelation drying, calcination
CS-9	Infiltration of aqueous solution of Sr-La-Fe salts and citric acid complexant to fresh not calcined Fe_2O_3 powder, gelation drying, calcination

Materials	Phase composition (XRD, wt%)/crystals size (nm)			Texture parameters (N_2-adsorption)		
	LSF	α-Fe_2O_3 hematite	γ-Fe_2O_3 maghemite	Surface area, m^2/g	Pore volume, cm^3/g	Average pore diameter, nm
Fe_2O_3	--	100/50	--	8	0.06	9.2
$La_{0.8}Sr_{0.2}FeO_3$	100/25	--	--	17	0.003	3
CS-1	29/25	56/40	15/20	16	0.1	13
CS-2	28/45	71.5/>50	--	13	0.1	23
CS-3	60/30	40/45	--	11	0.1	9
CS-4	58/30	36/45	6/15	10	0.1	10
CS-5	60/22	29/45	11/20	20	0.2	19
CS-6	57/45	43/45	--	14	0.1	20
CS-7	60/30	28/50	12/20	8	0.1	7
CS-8	61/25	13/45	26/25	12	0.1	8
CS-9	50/25	40/45	10/40	18	0.1	15

Table S2. The peak width measured in deconvoluted XPS spectra of as-prepared materials presented in Figure 11.

Materials	Lattice oxygen		Defect-affected oxygen		Surface oxygen		Carbonate and Organics oxygen		Oxygen in hydroxyl groups	
	B.E., eV	Width., eV	B.E., eV	Width., eV	B.E., eV	Width., eV	B.E., eV	Width., eV	B.E., eV	Width., eV
Fe_2O_3	529.6	1.0	-----	-----	530.7	1.3	----	-----	532.8	1.4
LSF	528.9	1.1	529.9	1.2	530.5	1.3	531.3	1.4	532.2	1.2
Core-shell CS-4	529.0	1.1	529.8	1.1	530.6	1.2	531.4	1.1	532.6	1.4
Core-shell CS-5	529.0	1.3	529.8	1.3	530.5	1.2	531.1	1.4	532.3	1.5

Table S3. Carbon species detected by XPS in as-prepared materials.

Materials	C1s: C-C/C-H			C1s: C-O-C			C1s: O-C=O		
	B.E., eV	% at.	Width., eV	B.E., eV	% at.	Width., eV	B.E., eV	% at.	Width., eV
Fe_2O_3	284.8	92.3	1.7	285.8	3.0	1.8	288.9	4.7	1.8
LSF	284.8	66.0	1.4	286.4	9.5	0.9	289.1	24.6	1.9
Core-shell CS-4	284.8	75.7	1.6	286.3	11.0	1.5	289.2	13.3	1.5
Core-shell CS-5	284.8	67.0	1.6	286.1	11.6	1.5	289.2	14.3	1.9

Table S4. The peak width measured in deconvoluted XPS spectra of materials recorded after H_2-TPR testing and presented in Figure S4.

Materials	Lattice oxygen		Defect-affected oxygen		Surface oxygen		Carbonate and organics oxygen		Oxygen in hydroxyl groups	
	B.E., eV	Width., eV	B.E., eV	Width., eV	B.E., eV	Width., eV	B.E., eV	Width., eV	B.E., eV	Width., eV
**LSF fresh**	528.9	1.1	529.9	1.2	530.5	1.3	531.3	1.4	532.2	1.2
H_2-TPR 450°C	-----	-----	530.2	1.9	-----	-----	531.9	2.0	533.7	2.0
H_2-TPR 650°C	-----	------	529.5	1.9	-----	------	531.7	2.0	532.7	2.0
H_2-TPR 870°C	528.7	2.0	-----	-----	-----	----	531.1	2.6	533.4	2.0
**CS-4 fresh**	529.0	1.1	529.8	1.1	530.6	1.2	531.4	1.1	532.6	1.4
H_2-TPR 450°C	528.3	2.1	529.6	2.3	-----	-----	531.0	3.0	-----	------
H_2-TPR 650°C	528.9	2.4	530.1	3.5	-----	------	531.6	4.3	-----	-------
H_2-TPR 870°C	529.2	2.2	----	-----	----	-----	531.0	2.1	532.7	2.4
**CS-5 fresh**	529.0	1.3	529.8	1.3	530.5	1.2	531.1	1.4	532.3	1.5
H_2-TPR 450°C	-----	-----	529.4	1.4	530.3	1.7	531.8	1.7	-----	-----
H_2-TPR 650°C	-----	-----	529.4	1.8	-----	-----	531.5	2.0	-----	------
H_2-TPR 870°C	-----	------	529.6	2.0	-----	------	531.4	2.2	533.0	2.2
**Fe_2O_3 fresh**	529.6	1.0	-----	------	530.7	1.3	----	------	532.8	1.4
H_2-TPR 450°C	-----	------	-----	-----	530.4	1.1	-----	------	532.6	1.8
H_2-TPR 650°C	------	-----	------	------	530.2	1.7	------	-------	532.6	3.4
H_2-TPR 870°C	-----	------	------	-----	530.3	1.8	------	-----	532.7	4.0

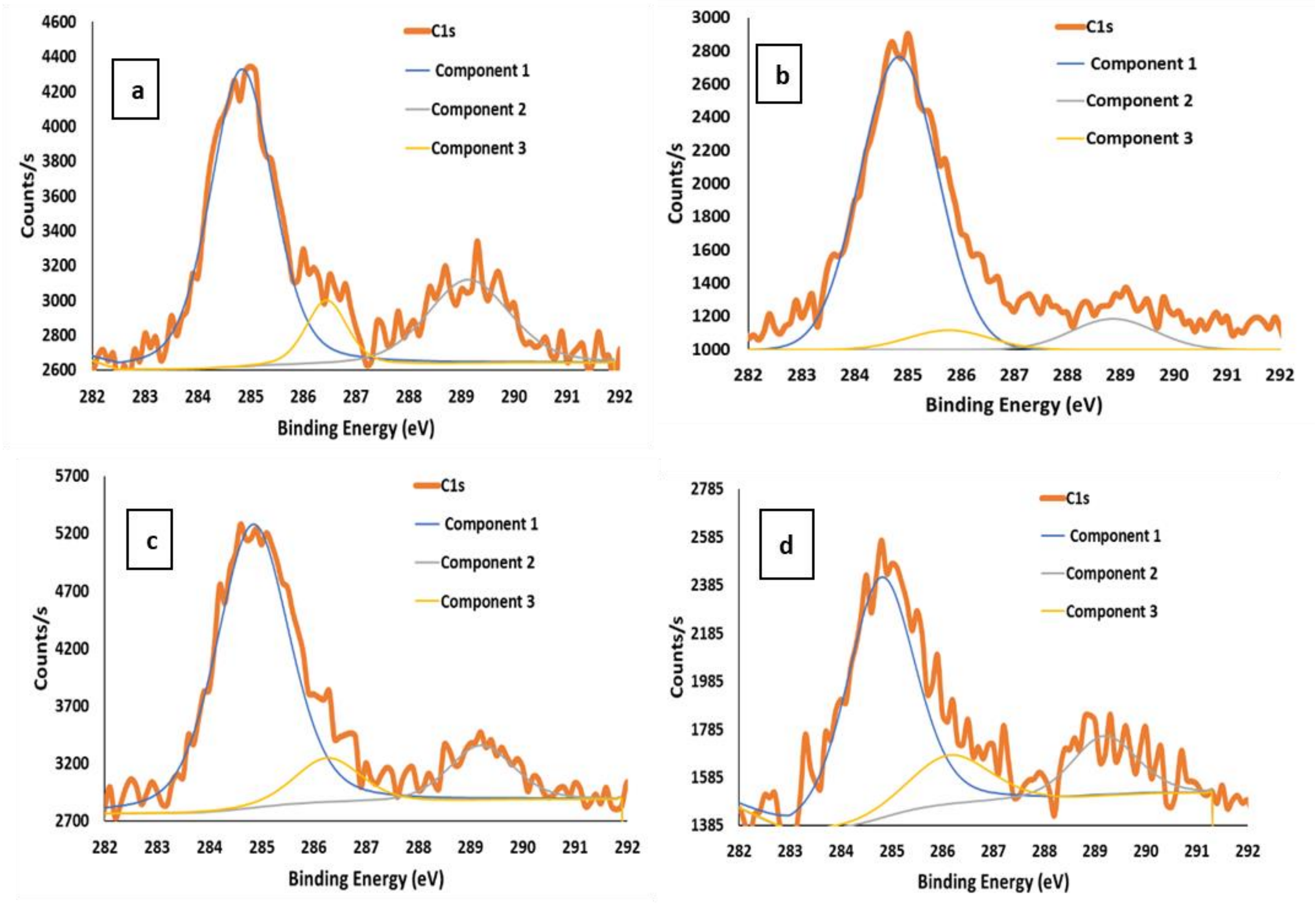

Figure S6. XPS spectra of C1s core: (a) LSF-perovskite; (b) Fe_2O_3 calcined at 700°C and Fe_2O_3@LSF core-shell materials - (c) CS-4 and (d) CS-5.

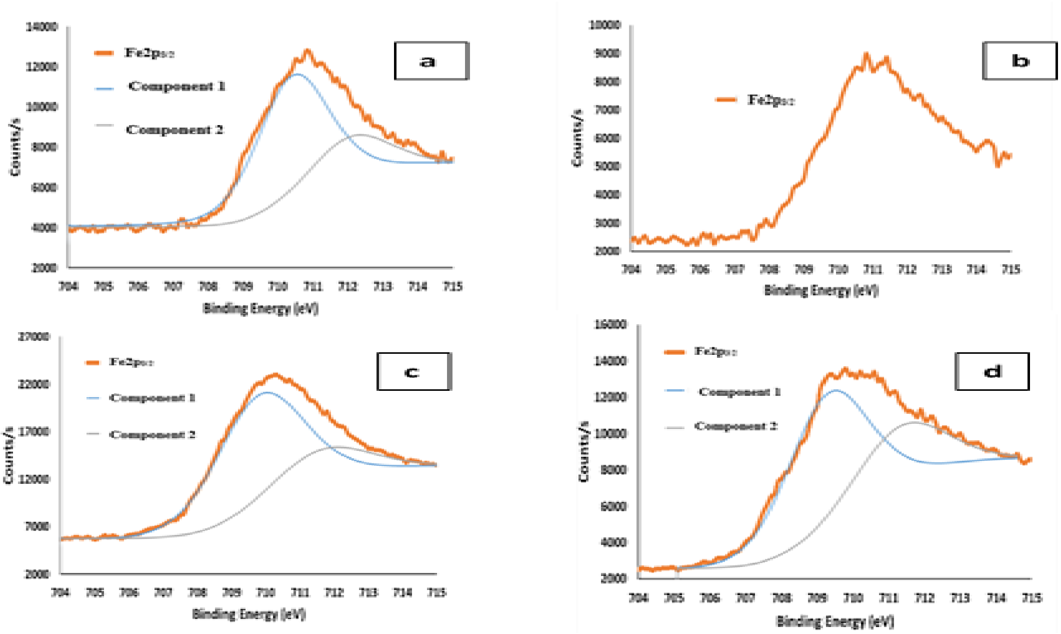

Figure S7. XPS spectra of Fe2p3/2 core: (a) LSF-perovskite; (b) Fe_2O_3 calcined at 700°C and Fe_2O_3@LSF core-shell materials - (c) CS-4 and (d) CS-5.

Table S5. Components of XPS spectra of Fe2p3/2 core recorded with as-prepared materials.

Materials	Fe2p3/2		Fe2p3/2	
	B.E., eV	% at.	B.E., eV	% at.
Fe_2O_3	711.3	100	-	-
LSF	710.4	73.3	712.1	26.7
Core-shell CS-4	710.7	78.2	712.1	21.8
Core-shell CS-5	710.6	66.1	712.2	33.9
